# Dissecting flowering time and flower color in *Carum carvi* utilizing a long-read draft genome and a GBS-based QTL mapping

**DOI:** 10.1038/s41598-026-61767-1

**Published:** 2026-07-14

**Authors:** Daniel von Maydell, Fang-Shiang Lim, Martin Junghanns, Yvonne Poeschl, Holger Budahn, Frank Marthe, Jens Keilwagen, Thomas Schmutzer

**Affiliations:** 1 Institute for Breeding Research On Horticultural Crops, Julius Kühn Institute, Quedlinburg, Germany; 2 Institute for Biosafety in Plant Biotechnology, Julius Kühn Institute, Quedlinburg, Germany; 3 Dr. Junghanns GmbH, OT Groß-Schierstedt, Aschersleben, Germany; 4 Institute of Agricultural and Nutritional Sciences, Chair of Plant Breeding, Martin Luther University Halle-Wittenberg, Halle, Germany

**Keywords:** Flowering regulation, Genetic map, Linkage, Quantitative trait locus, Umbelliferae, Vernalization, Biotechnology, Genetics, Plant sciences

## Abstract

Caraway (*Carum carvi* L.) is a major essential oil crop with biennial and annual flowering types. As basic research resource, we developed a draft genome assembly using long-read ONT sequencing and provide a structural and functional gene annotation for an annual caraway inbred line. To elucidate the genetic control of flowering, a genotyping-by-sequencing (GBS) was conducted for an F_2_ population (N = 187) generating 731 strictly filtered SNPs. A linkage map was constructed spanning 663 cM across 10 linkage groups with in total 634 (full map) or 259 (thinned map) SNPs. Contrary to its dominance in F_1_, annual flowering occurred in only 36% of F_2_ plants under late sowing conditions. Furthermore, the annual F_2_ plants exhibited delayed flowering compared to the annual parent. QTL analysis identified five significant QTLs for (adjusted) flowering time (LG02, LG03, LG05, LG08 and LG10) explaining 6.1% to 10.5% (in total 42.7%) of phenotypic variance. The results support a polygenic predominately additive model for flowering induction in caraway. In addition, two QTLs for flower color (LG01, LG10) were detected explaining 10.9% and 26.0% of phenotypic variance, respectively. This study provides a comprehensive genomic resource for caraway, bridging the gap between traditional breeding and molecular improvement.

**Supplementary Information:**

The online version contains supplementary material available at 10.1038/s41598-026-61767-1.

## Introduction

Caraway (*Carum carvi* L., 2n = 2x = 20) is a key essential oil crop of the Apiaceae family, with an estimated genome size of 2.14 pg/1C^1^. It occurs in two distinct flowering types: biennial and annual^2^. Biennials require a cold stimulus (vernalization) once they have reached a juvenile stage of at least six leaves and a rootstock diameter of 5 mm before winter to initiate flowering^3^. Depending on growing area, biennials need to be sown from May to end of June to reach that size^3^, meaning they occupy the field for two growing seasons. In contrast, annuals complete their life cycle within a single year. While annuals are typically sown in spring, they can also be grown as "winter-annuals" (autumn sowing) to achieve higher grain yields by utilizing early spring moisture and a prolonged vegetation period. However, this cultivation system requires a high degree of winter-hardiness, a trait currently found in only one commercial annual variety (‘Aprim’, Agritec Ltd.).

In general, genetic diversity is limited in the annual genepool, which is thought to have originated from North Africa^4^. In contrast, the biennial genepool is widespread across Eurasia and offers a vast reservoir of genetic variation for agronomic traits, quality, and environmental adaptation. To enrich the annual genepool, introgression from biennials is a promising breeding strategy. While crossbreeding is possible through synchronized flowering by artificial vernalization, fixation of an annual flowering habit and early flowering, which is associated with higher yields^5^, remains challenging. Flowering type in caraway is reported to be controlled by a major locus, with annual flowering habit being dominant^3,6^. This dominance allows the recessive biennial allele to persist undetected throughout the breeding process. Further on, Nemeth and Pluhar^6^ hypothesized that several minor loci of biennials may decelerate flowering time.

In a screening of various annual and biennial genotypes, few exceptionally rose-flowering biennial genotypes were detected^7^. Although not a primary trait for field production, rose-flowering could increase the economic potential of caraway as an ornamental plant. Since these genotypes are small wild types, an introgression of the rose-flowering trait into cultivated material is necessary for economic use.

Identifying diagnostic genetic markers for flowering type, flowering time, and flower color could aid in efficiently screening early breeding generations to fix annual, early-flowering, or rose-flowering habits. So far, single nucleotide polymorphism (SNP)-based diagnostic (PACE/KASP) markers have been developed from a diversity panel of annual and biennial flowering types^8^. These markers have been proven useful for checking crossing success in various crosses between annuals and biennials, but cannot reliably predict the flowering habit. Bocianowski and Seidler-Lozykowska^9^ reported significant associations for several quantitative traits using randomly amplified polymorphic DNA (RAPD) markers, but did not include flowering time. In general, the genetic dissection of traits in caraway is hampered by a lack of physical sequence information (a genome assembly) and a genetic map.

In this study, we aimed to address these limitations by:

1. Detecting quantitative trait loci (QTL) affecting flowering type, flowering time, and flower color based on a biparental F_2_ mapping population utilizing genotyping-by-sequencing (GBS) data. Meanwhile, developing a genetic map to bring genetic markers into an order.
2. Introducing an annotated draft genome assembly of caraway based on long-read sequencing data and using this assembly for efficient mapping of GBS data as well as for the genetic dissection of detected QTL regions to identify candidate genes.

## Material and methods

### Construction of a draft genome assembly & annotation

#### DNA extraction and purification

In total, 2 g of leaf material was taken from the annual S_7_ inbred line ‘WS49’ (JKI). This line was chosen because of increased homozygosity by inbreeding and its key role within summer-annual and winter-annual breeding programs, notably carrying genetic determinants for winter-hardiness. Thus, ‘WS49’ provides a robust reference suitable for broad genetic applications beyond the scope of this study. For high molecular weight (HMW) DNA extraction the gravity column based NucleoBond HMW DNA kit from Macherey–Nagel was used. We then enriched long DNA fragments by applying the Short Read Eliminator (SRE) XS kit from PacBio. For quality check, DNA quantification was done using the Qubit dsDNA BR Assay-Kit on the Qubit fluorometer. Concentration after HMW DNA extraction was measured at 556 ng/µl and after SRE a concentration of 137 ng/µl was achieved.

#### Genome sequencing

The sequencing library was constructed using the SQK-LSK114 kit from Oxford Nanopore Technologies. Purified HMW DNA was used and 2 µl with 20 fmol were loaded onto a PromethION flow cell (FLO-PRO114M). Sequencing was performed on a PromethION24 system using in total three flow cells running for 72 h each (Figure S1 shows overall flow cell performance over run time). Base calling was run in SUP accuracy mode with 400 bps using Guppy (version 6.4.6) and a minimal length of 200 nucleotides was required. The three flow cells reached raw read N50 values (23.47 kb, 25.48 kb and 25.35 kb) and generated a total of 321.97 Gb (67.43 Gb, 110.73 Gb and 143.81 Gb). With 298.63 Gb, 92.8% of all reads passed a min Q score of 10. Descriptive statistical reports were generated with toulligQC (https://github.com/GenomiqueENS/toulligQC) which stated high-quality of the sequencing process.

#### Genome assembly and performance statistics

To construct the draft genome of caraway the Flye assembler from Kolmogorov, et al^10^. was used in version 2.9.1. The genome size of caraway was estimated at 2.1 Gbp based on flow cytometric data of a caraway germplasm collection as previously reported^1^. This value was used as the genome size parameter for the Flye assembly. Before use, ONT reads were screened for contamination, adapters were removed and reads were required to have an average high quality (Q > 20). In the genome assembly process standard parameters were used except ‘min_overlap’ set to 7,000 bp, the ‘min_read_length’ set to 21.5 kb and the coverage of the initial disjointing process was set to ‘40’. The constructed genome graph was inspected using GfaViz^11^. For a comprehensive assessment of the genome assembly, we measure the three relevance levels contiguity, completeness and correctness using an ensemble of bioinformatic methods.

The taxonomic classification and profiling of the constructed assembly were performed using KRAKEN2 in the version 2.1.3^12^. The analysis was based on a k-mer concept using a subset of the nt database for bacteria, fungi, human, plant, and virus genomes. To assess the completeness of the assembly, the Benchmarking Universal Single-Copy Orthologs (BUSCO, version 5.8) software was utilized by screening the conserved eudicotyledons_odb12 gene set, which comprises of 2,805 orthologous genes from 76 genomes^13,14^. Quantitative measures were provided for the classes: complete, duplicated, fragmented, and missing. Furthermore, we utilized the long read-based Inspector software in version 1.3.1^15^ to evaluate the assembly and to identify potential regions of assembly errors.

#### Annotation

Structural gene annotation was predicted using a combination of ab initio and homology-based gene prediction. Ab initio gene prediction was performed with Tiberius v1.1.8 using the plant-trained model eudicotyledons.yaml^16^. Homology-based gene prediction was then performed with GeMoMa v1.9^17–19^, using annotations from the following reference species: *Arabidopsis thaliana* TAIR10 (GCF_000001735.4)^20^, *Daucus carota* v3.0 (GCF_001625215.2)^21^, and *Coriandrum sativum* (http://cgdb.bio2db.com/; accessed 17 January 2026)^22,23^. The Tiberius gene predictions were supplied to GeMoMa as external predictions. Default parameters were used, except that the GAF filter expression was replaced with start = = 'M' and stop = = '*' and (evidence > 1 or (!isNaN(pAA) and pAA > = 0.7)) to retain high-confidence gene models. Similar to the assessment of genome, annotation quality was subsequently assessed with BUSCO using the eudicotyledons_odb12 lineage^13,14^ and OMark using the Viridiplantae database^24^. The resulting gene models were functionally annotated using InterProScan v5.59–91.0 with the parameters -f TSV -applications TIGRFAM, FunFam, SFLD, PANTHER, Gene3D, Hamap, PrositeProfiles, SMART, CDD, PRINTS, PIRSR, PrositePatterns, AntiFam, Pfam, PIRSF -pathways -goterms^25^.

### Biparental mapping population

**Fig. 1:**
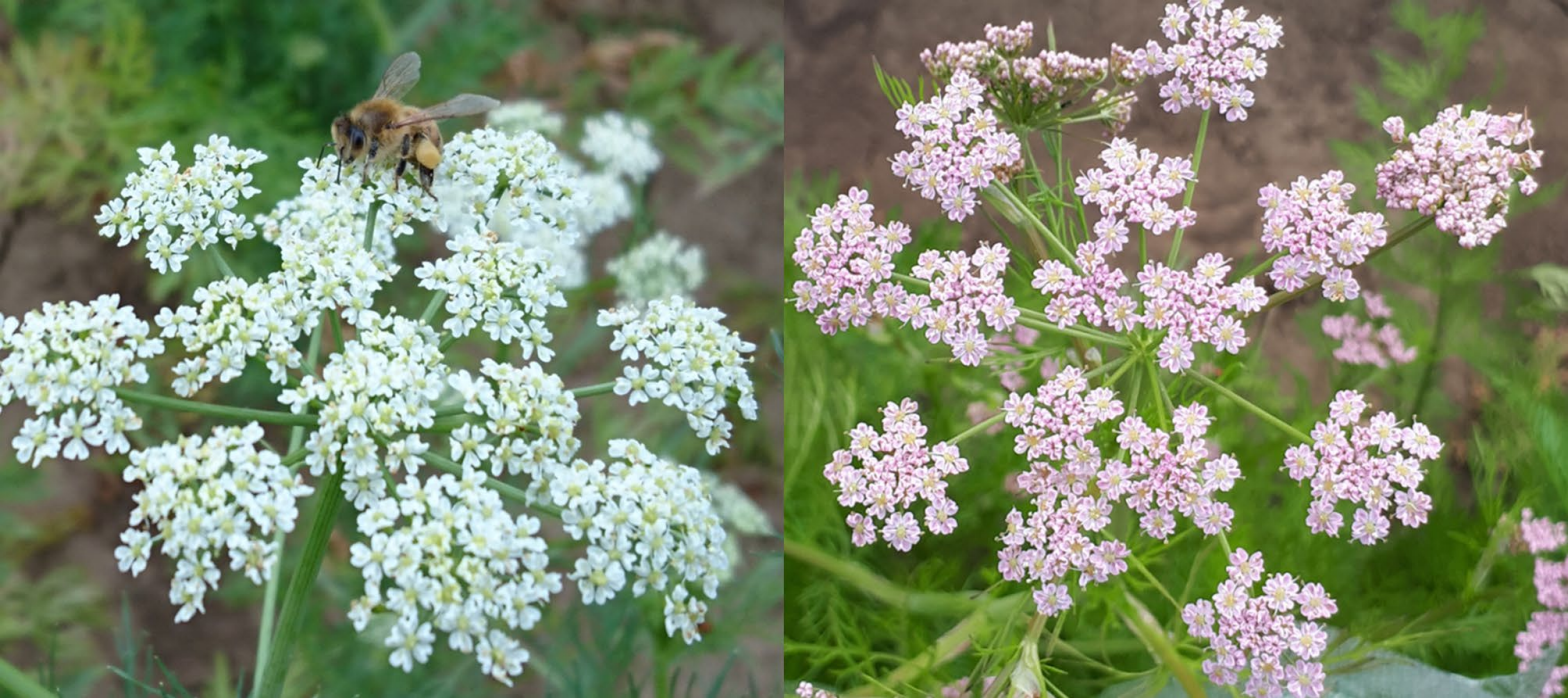
Phenotypic differences between parental plants. The annual inbred line “L32” (left) shows white flowers, while the biennial accession CARUM 63 (right) displays rose-colored flowers.

One plant of the biennial wild accession CARUM 63 from the Leibniz Institute of Plant Genetics and Crop Plant Research (IPK, Seeland OT Gatersleben, Germany) was crossed with one plant of the annual inbred line “L32” of the JKI of the 5th inbred generation. This inbred line displays the standard white flower color of caraway, while the biennial accession shows a prominent rose flower color (see Fig. 1). Both, homogeneous biennial flowering and rose flower color has been determined in a two-year field trial (accession ID here: Cc42_RUS_wd)^7^. The taxonomic identity of CARUM 63 was verified by the curators of the IPK genebank, and reference herbarium specimens for the IPK caraway collection are maintained at the Herbarium Gatersleben (GAT). The biennial accession was once donated by the botanical garden in Halle (Saale), Germany, but originates from Russia (according to passport data from IPK). This accession was also chosen because it was late flowering among various biennial accessions, which might allow the detection of additional QTLs for flowering time^7^. One F_1_ plant from the cross was self-fertilized to produce a segregating F_2_ mapping population. Because the biennial parent retained residual heterozygosity, the use of a single F_1_ founder ensured that the F_2_ population segregated from a defined set of parental haplotypes. While the use of multiple independent F_1_ individuals could potentially capture additional haplotype combinations and recombination events, it would also increase genetic heterogeneity within the mapping population. The present design therefore provides a genetically well-defined biparental population suitable for linkage and QTL mapping.

### Phenotyping

**Fig. 2:**
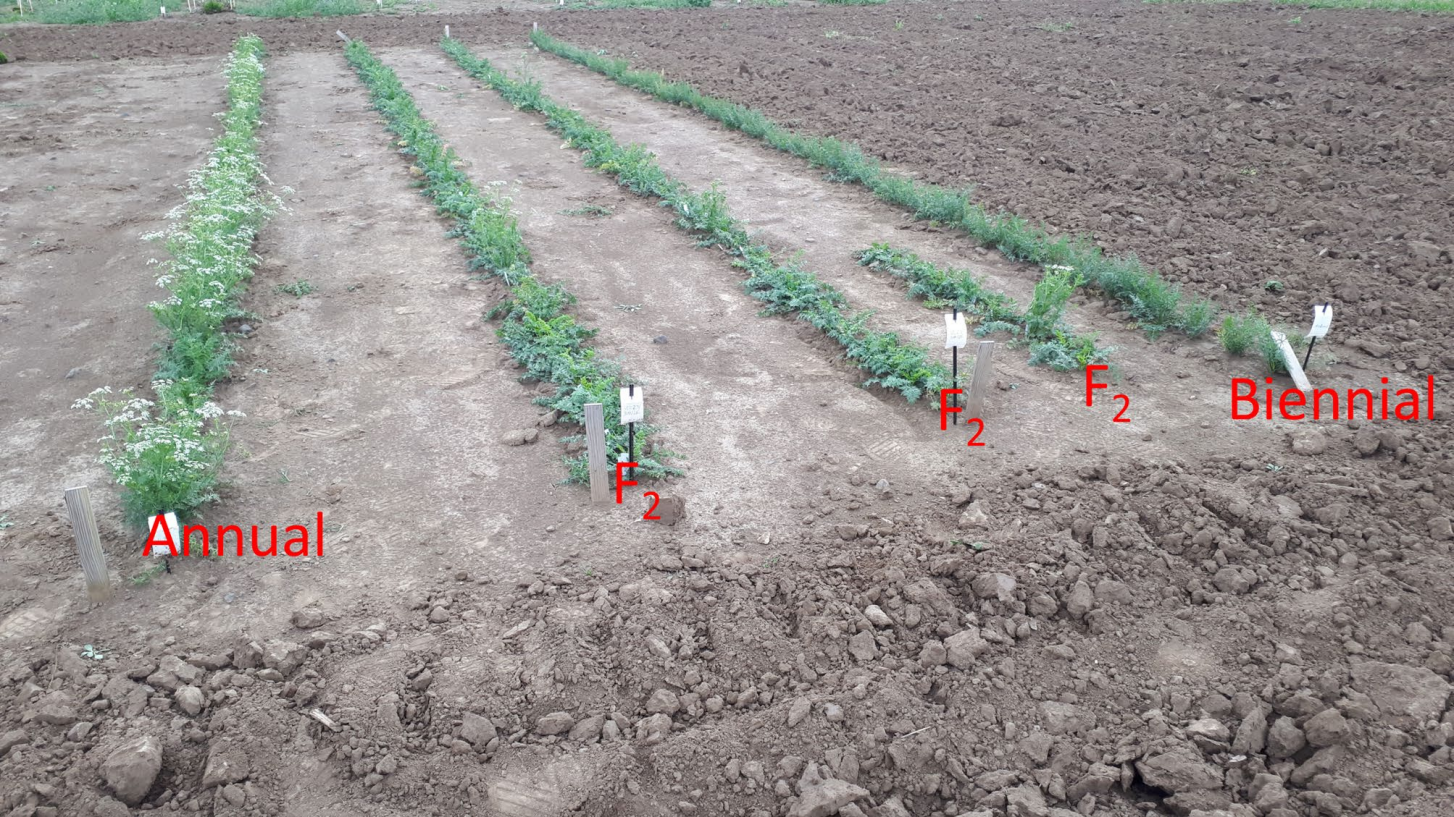
Field trial in September 2023. Row 1 (from the left) shows the annual control with white flowers in full bloom. Rows 2–4 represent the still sparsely flowering F_2_ mapping population, and row 5 contains the non-flowering biennial control.

Seeds of F_2_ and controls were sown and seedlings were pre-grown in the greenhouse in May 2023. In June, pre-grown plants were transplanted to a field trial consisting of five rows, with 10 cm spacing between plants and 100 cm between rows. As controls, 90 annual plants and 90 biennial plants were planted in rows 1 and 5, respectively. Since the biennial parent did not produce sufficient seeds by self-fertilization, the cultivar ‘Rekord’ was used as phenotypic biennial control during the field trial. In total 187 F_2_ plants were grown in the intermediate rows 2 to 4 (see Fig. 2).

For each F_2_ plant, flowering time was recorded as the number of days from sowing until the beginning of flowering, defined as the emergence of the first anthers (BOF1). To account for the gap between annual and biennial flowering, a second measure was calculated (BOF2) by subtracting a fixed period of 145 days without new flowering individuals (“winter period”) from the values of biennial plants. The adjustment value of 145 days used to calculate BOF2 was derived directly from the observed vegetative winter gap in the field. The final annual individual flowered 161 days after sowing (November 08, 2023), whereas the earliest biennial individual initiated flowering 307 days after sowing (April 02, 2024), revealing a 146-day interval entirely devoid of flowering events. This reduced the bimodal distribution of the data to improve the robustness of the used parametric models. In addition, BOF2 accounts for the environmental truncation under field conditions, acknowledging that several genotypes with a potential annual flowering habit were physically prevented from flowering by the onset of winter due to the late sowing date, which is one reasonable explanation for the observed segregation ration in F_2_ (see discussion). To search for flowering time loci independent from flowering type, a standardized beginning of flowering (BOF3) was calculated by Z-transforming BOF1 separately for annual and biennial flowering types. Through this transformation, the resulting BOF3 value no longer reflects the absolute time to flowering, but rather how early or late an individual plant flowers relative to its flowering type. This effectively scales down the massive genetic effect of the annual/biennial switch to zero, enabling the downstream mapping analysis to detect genetic loci that modulate the timing of flowering within both groups. As pivotal advantage to two separate MQM analyses, a moderate sample size is retained. In addition, flowering time was simplified recorded as flowering type, which differentiated between annual (i.e. flowering before winter) and biennial flowering types (i.e. flowering after winter; FT1). In contrast to BOF1-3, FT1 also includes plants, which died during winter. These plants, which showed a healthy condition before winter but were predominately damaged by mouse were regarded as biennial plants to avoid selection bias against biennial genotypes.

We conducted a chi-square test for goodness-of-fit using the function chisq.test in R^26^.

Flower color was scored (rose or white) at an early flowering stage, when at least one umbel had fully developed petals. All plants showing a rose coloration (disregarding color intensity) were defined as rose-flowering. Although strong variation in rose color intensity was observed, preliminary field observations indicated that pigmentation intensity was strongly influenced by environmental conditions, particularly pigment bleaching under high light exposure. As a consequence, quantitative scoring of color intensity under field conditions was considered insufficiently robust and reproducible. The objective of this study was therefore not to quantify pigmentation intensity but to distinguish between the presence and absence of anthocyanin-derived flower coloration. Collapsing the phenotype into a binary classification reduced environmentally induced variation and enabled consistent scoring across all individuals for subsequent genetic analyses.

An overview of all trait descriptors is presented in Table 1 (result section).

**Table 1:** Description of phenotypic traits and scoring methods for flowering time and flower color. Abbr. = abbreviation; N = sample size.

Abbr	Definition	Values	N
BOF1	Beginning of flowering (first anthers visible) in days after sowing. [One outlier (F133: BOF1 = 178; |Z|> 3 within annuals) during mapping and for calculating BOF2/BOF3 removed.]	106—356	163
BOF2	Beginning of flowering (first anthers visible) in adjusted days after sowing; adjusted by subtracting 145 days for biennial plants (winter period without new flowering plants) from BOF1	106—234	163
BOF3	Standardized beginning of flowering (Z-score); calculated by Z-transforming BOF1 separately for annual and biennial flowering types	−1.55 – 2.68	163
FT1	Flowering type, differentiating between annual and biennial flowering (including plants died over winter as biennial flowering types)	0 = annual 2 = biennial	180
COL	Flower color	0 = white 2 = rose	164

### Genotyping by sequencing, SNP discovery and filtering

DNA was extracted from young developing leaves using DNeasy Plant Mini Kit (Qiagen, Venlo, Netherlands). DNA quality and quantity were tested using Nanodrop 8000 and Qubit Flex Fluorometer using dsDNA Assay BR (all Thermo Fisher Scientific, Waltham, USA) and gel electrophoresis. Library preparation for GBS was carried out at the Institute of Integrative Biology and Systems (IBIS) of University Laval (Quebec, Canada). Genomic DNA was normalized to 10 ng/µl and digested with the restriction enzymes PstI and MspI^27^. Libraries were sequenced using an Illumina NovaSeq 6000 platform at Génome Québec (Montréal, Canada), generating 150-bp paired-end reads.

Raw sequences were processed and SNP calling was performed using the internal Galaxy server (version 25.0) within JKI^28^. First, GBS raw reads were trimmed using Trim Galore v0.6.7; non-default parameters: Phred quality score threshold for low-quality ends: 30; discard reads shorter than 50 nucleotides^29^;. Trimmed reads were mapped against a draft assembly (2171 contigs, see below) using BWA MEM v0.7.18^30,31^. SNP calling was carried out using bcftools mpileup and bcftools call v1.15.1 (non-default-parameters: output-tags DP, DPR)^32^.

SNPs were filtered in three steps: (i) quality filters (minimum quality score ≥ 40, minimum read depth per SNP = 4) to eliminate low-confidence base calls while minimizing the loss of informative loci; (ii) selection of SNPs homozygous and polymorphic between parental genotypes; (iii) additional filters (≤ 10% missing data, 35–65% heterozygosity, and 15–35% representation of both homozygous genotypes in mapping population). These stringent downstream thresholds were applied to rigorously purge technical noise, thereby ensuring a structurally sound framework for de novo linkage map construction.

### Construction of a linkage map and QTL mapping

A linkage map was constructed using the software JoinMap 5.0 using default settings^33^. Markers were grouped using a LOD threshold of 4 (no changes were observed up to a LOD of 10), and regression mapping with Kosambi’s mapping function was applied. Loci with mean χ^2^ contributions > 5 were removed. To ensure model stability and minimize multicollinearity during QTL mapping, the genetic map was thinned to a maximum density of one marker per 1 cM. In clusters of tightly linked markers, the marker with the lowest proportion of missing data was chosen to represent the bin.

QTL analysis was performed using MapQTL 5^34^. Genome-wide LOD significance thresholds (α = 0.05) for each trait were determined by 1,000 permutation tests. Initially, interval mapping (IM) was used to identify candidate QTL regions. To refine QTL positions and to detect loci potentially masked by major-effect QTL, multiple QTL mapping (MQM) was subsequently applied. To ensure model parsimony, only cofactors improving the explained variance at significant QTLs were retained in the final MQM model (final cofactor settings per trait can be found in Table S1F). Final QTL positions, effects, and explained variance were estimated from the MQM model, whereas 2-LOD- and 1-LOD support intervals were derived from a restricted MQM (rMQM). Markers immediately outside the intervals were defined as flanking markers. To validate QTLs for the non-parametrically distributed traits (COL, FT1), a Kruskal–Wallis rank sum test was performed.

Although BLINK was originally developed for association mapping, the Bayesian-information and Linkage-disequilibrium Iteratively Nested Keyway (BLINK) model^35^ was used as implemented in the R package GAPIT^36^ to cross-validate MQM peaks. Unlike single-locus models (MLM/GLM), BLINK’s multi-locus approach effectively eliminated redundant signals caused by high local LD. BLINK was performed using the entire set of 731 filtered SNPs. This allowed for a higher-resolution scan of the genome, leveraging the full allelic information available in our GBS data. To accommodate the high number of underlying contigs within the software’s architecture, markers were assigned to a single continuous linkage group and numbered consecutively.

## Results

### Draft genome assembly & annotation basic statistics

#### Draft genome assembly

**Fig. 3:**
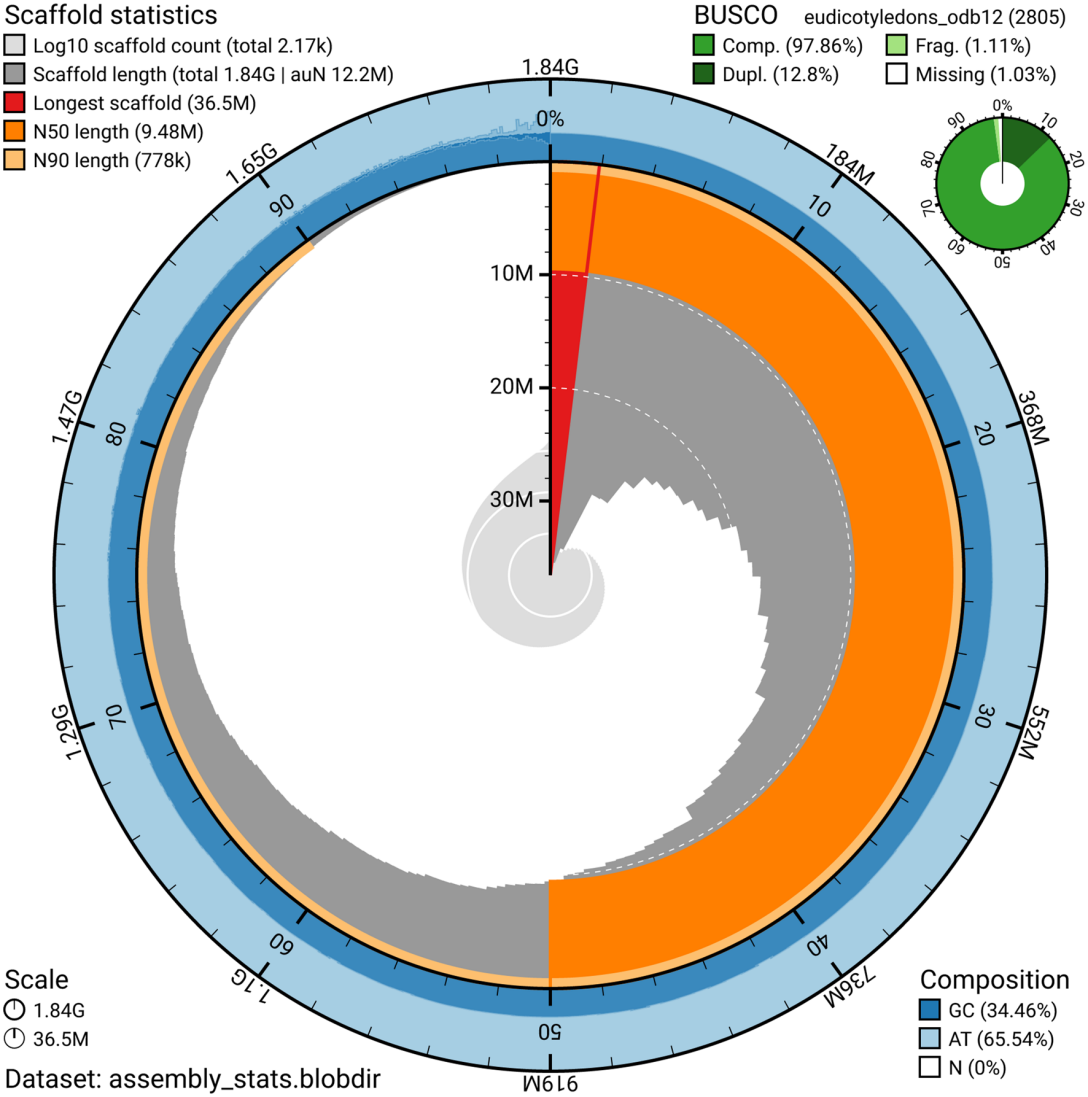
Snail plot. Summarizes the main assembly metrics for the 1.87 Gbp draft genome.

The constructed draft assembly of caraway reached a total size of 1.87 Gbp consisting of 2,171 fragments. The assembly exhibited high contiguity with a contig N50 of 9.5 Mb, and 820 scaffolds were found to be larger than 100 kb in length. Taxonomic profiling via KRAKEN2 revealed that the vast majority of the constructed contigs were associated with plants, indicating low contamination rates (Table S2). Only a minor fraction of three contigs (0.14%) remained unclassified. The BUSCO assessment confirmed a high level of genomic completeness (97.9%, Table S3). Detailed contiguity and completeness metrics are further summarized in the snail plot (Fig. 3). Additionally, the final evaluation with Inspector confirmed the quality of the assembly by an overall mapping rate of 99.74% and a depth in large contigs of 158.92.

#### Annotation

**Fig. 4:**
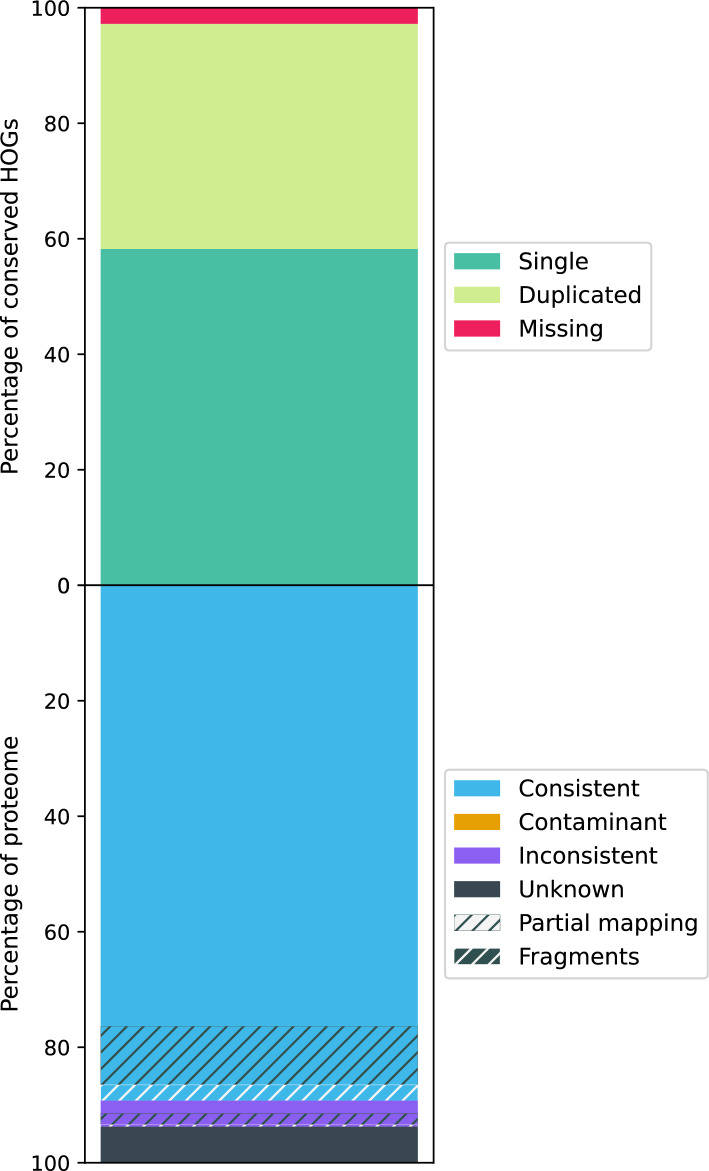
Proteome completeness and consistency. Results from the OMARK assessment for the *Carum carvi* draft assembly.

Structural gene prediction yielded 41,835 genes and 51,645 transcripts. Quality assessment of the structural gene annotation using BUSCO indicated comparable results for Carum carvi, Daucus carota and Arabidopsis thaliana, and much better results than Coriandrum sativum (Table S3). Furthermore, BUSCO results for genome and annotation were highly similar, with slightly better results for the annotation of 98.6% completeness. Additionally, quality assessment with Omark indicated that structural gene prediction was of high quality, with the majority of predictions being highly consistent and only a few being missing, inconsistent or partial (Fig. 4).

### Phenotypic data

#### Flowering time and flowering type

F_1_: Prior to the establishment of the F_2_ mapping population, phenotypic attributes of the F_1_ generation were assessed under controlled greenhouse conditions. While finally a single F_1_ plant was selected to produce the F_2_ seeds utilized in this study, notably, all cultivated F_1_ individuals initiated flowering without requiring a vernalization period, indicating that the annual flowering habit is qualitatively dominant over the biennial trait. However, a considerable developmental delay in flowering time was observed in the F_1_ plants compared to annual parental genotypes grown simultaneously under identical conditions.

F_2_ and controls (field trial): All 90 plants of the annual control had initiated flowering by mid-September 2023, approximately 106 days after sowing. In contrast, none of the biennial control plants flowered during the first year. Among the 187 cultivated F₂ plants, 23 plants died before initiating flowering. Seven and 16 of these plants died before or during winter, respectively. These 16 plants were regarded as biennial flowering types (FT1), but were disregarded for quantitative trait descriptors (BOF1, BOF2, BOF3). Consequently, flowering type was assessed for 180 plants, while other traits descriptors were obtained for 164 plants (Table 1).

**Fig. 5:**
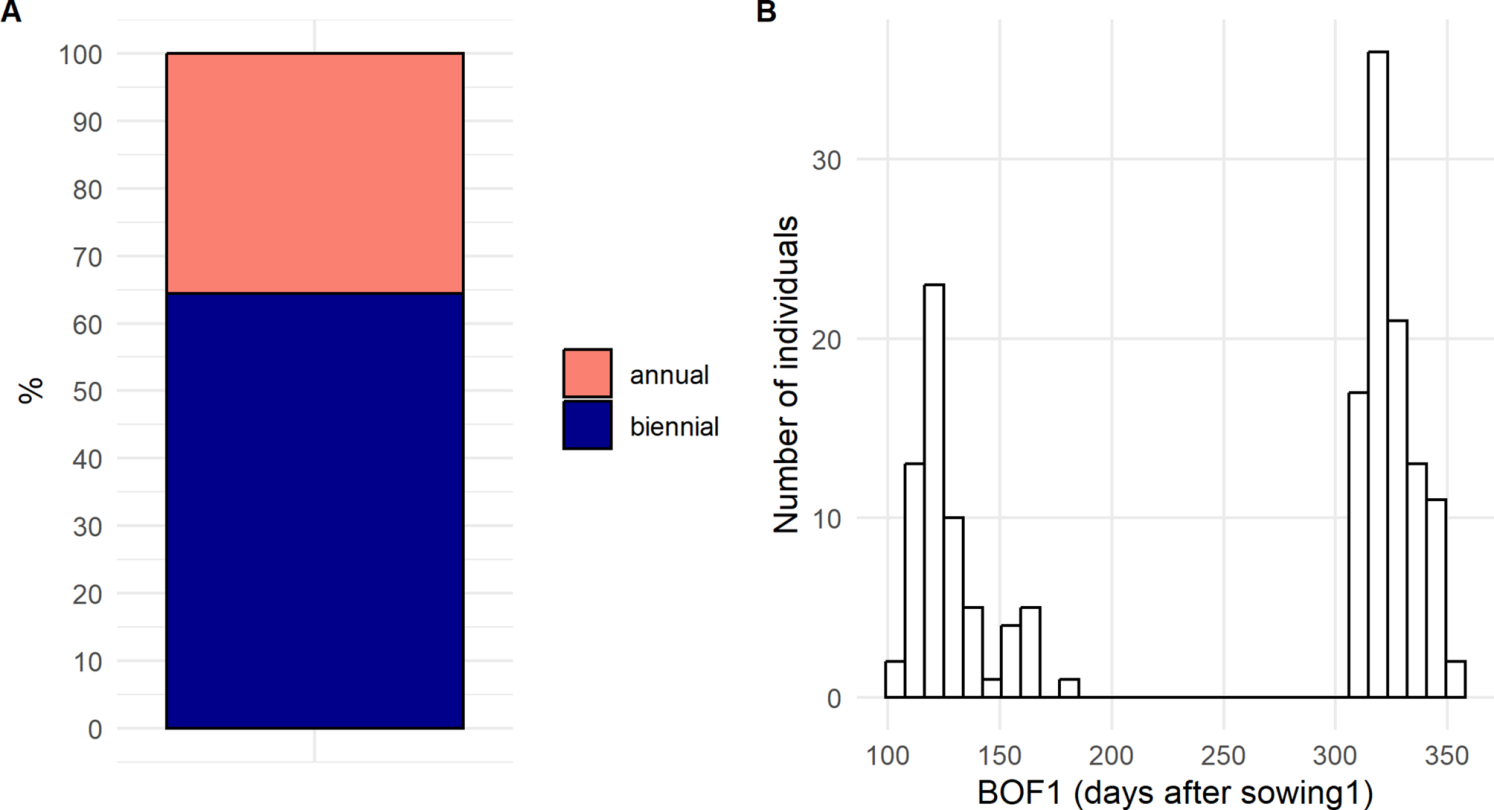
Flowering time. (**A**) Percentage of annual (red) and biennial (blue) flowering types among 180 F₂ plants. (**B**) Variation in beginning of flowering (BOF1) in days after sowing, recorded for 164 F₂ plants.

Overall, 36% (64 plants) were classified as annual flowering types and 64% (116 plants) as biennial flowering types (Fig. 5A). For annual plants, the beginning of flowering (BOF1) ranged from 106 days after sowing (September 14, 2023) to 161 days after sowing (November 08, 2023). One outlier (178 days after sowing; November 25, 2023) was removed. For biennial plants, the beginning of flowering ranged from 307 days after sowing (April 02, 2024) to 356 days after sowing (May 21, 2024) (Fig. 5B). BOF1 was used to compute BOF2 and BOF3 (see material & methods and Table 1) for mapping purposes. All phenotypic data are deposited in Table S1A.

We formally tested the observed segregation against a 3:1 ratio (annual:biennial according to Nemeth and Pluhar^6^) using chi-square test for goodness-of-fit, which was rejected: χ2 = 149.36, p < 0.001.

#### Flower color

**Fig. 6:**
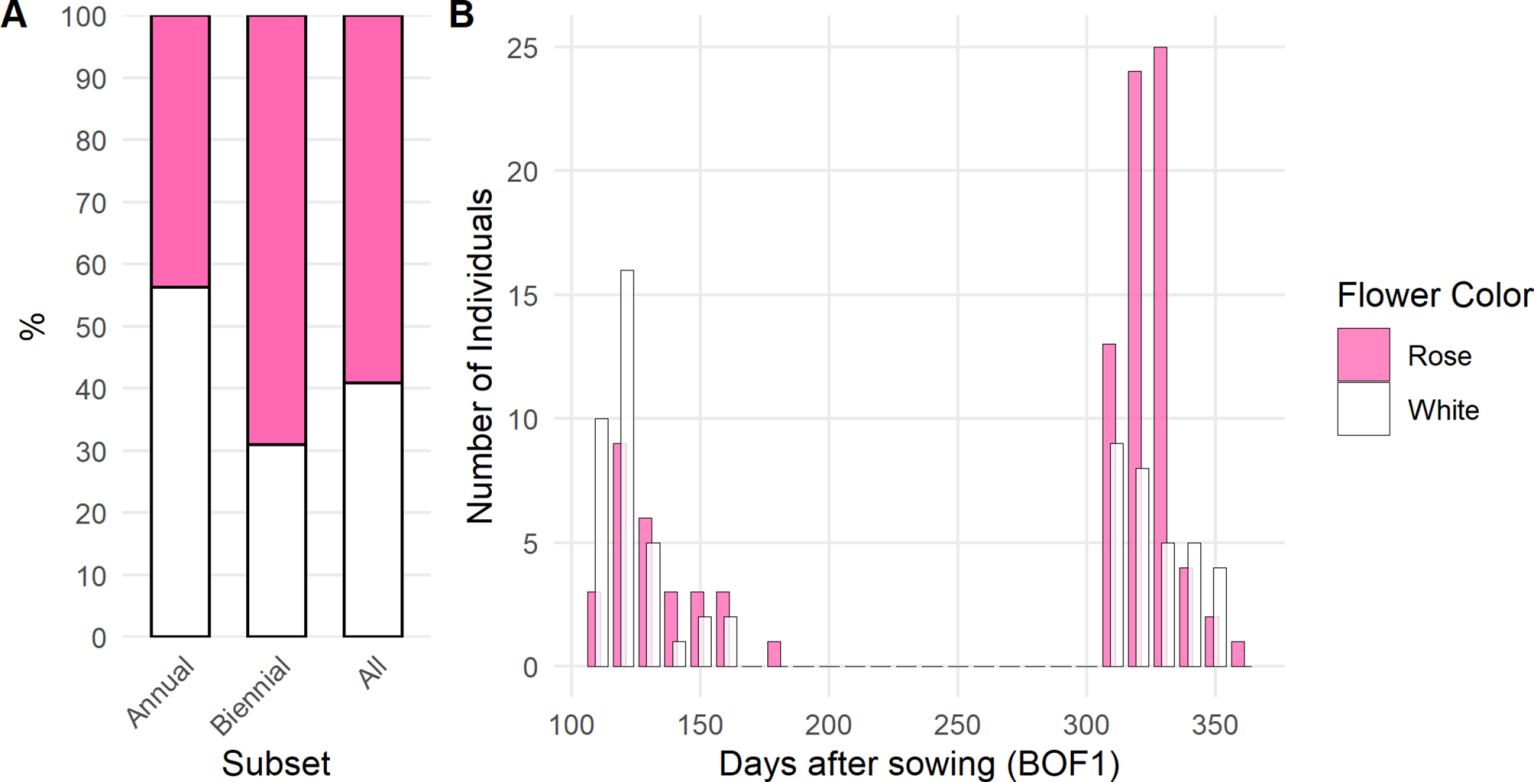
Flower color. (**A**) Relative frequency (%) of rose and white flowering phenotypes, stratified by annual (n = 64) and biennial (n = 100) flowering types. (**B**) Variation in the beginning of flowering (BOF1, days after sowing) for rose and white flowering individuals.

Under greenhouse conditions during crossings and F_2_ production, we observed that the biennial parent exhibit a strongly bleached rose coloration. In the field trial, we observed a wide range of flower colors in F_2_ plants, ranging from white and light rose to deep rose (as in Fig. 1). Due to the continuous range and noticeable bleaching on sunny days, we did not define intermediate flower colors. Thus, all plants showing a rose coloration were defined as rose-flowering. In total, we detected 59.1% rose-flowering plants, while biennial flowering types had a higher percentage of 69.0% (Table 2, Fig. 6A). In more detail, for early-flowering annuals and late-flowering biennials, a higher proportion of white-flowering plants was observed in the F_2_ generation (Fig. 6B).

**Table 2:** Distribution of flower color phenotypes (rose vs. white) among annual and biennial flowering types in the mapping population (n = 164). Frequencies are provided as absolute counts and relative percentages (%).

**COL**	**Count**			**Percentage**		
	Annual	Biennial	All	Annual	Biennial	All
Rose	28	69	97	43.8	69.0	59.1
White	36	31	67	56.2	31.0	40.9
Sum	64	100	164

### Genotyping by sequencing, mapping and filtering

In total 2,249 million sequences were gained by GBS for all 187 F_2_ plants and parental plants after trimming. The number of sequences per sample ranged from 0.8 to 32.8 million sequences and averaged 11.9 million. On average 95.6% of the sequences were mapped to the draft genome assembly. 97.5% and 99.4% of the sequences of the annual and of the biennial parent were mapped to the draft genome, respectively. After a first quality filter 9,787 SNPs were gained by GBS. While the biennial parent showed a heterozygosity of 24.81%, the annual parent had a heterozygosity of 5.92%. After filtering for homozygous SNPs between parents and the segregation ratio, 731 SNPs remained (Table S1B), which were used for linkage mapping in Joinmap. The full filtering pipeline and the effect of each filter (including intermediate steps) on SNP number is reported in Table S1B.

### Linkage map

**Fig. 7:**
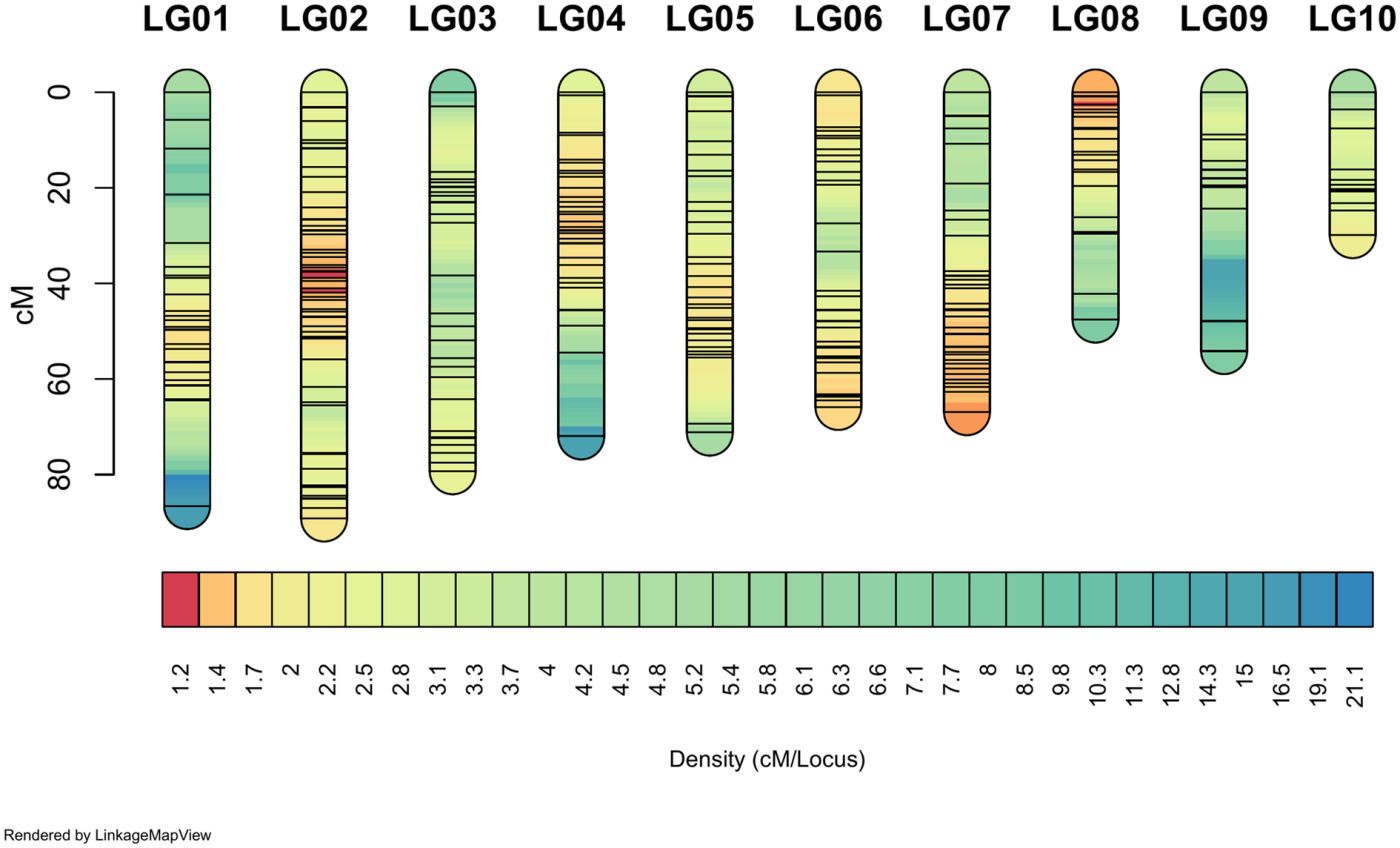
Thinned linkage map. Density distribution of SNP markers across the 10 linkage groups (LG) of the thinned genetic map comprising 259 loci.

Markers were grouped into 10 linkage groups. From the 731 initial SNPs, 634 SNPs were mapped by regression mapping (full map in Table S1C). A thinning to one marker per cM resulted in a final map of 259 markers spanning a total of 663 cM across 10 LGs (thinned map in Table 3, Table S1D). The number of SNPs per LG ranged from 11 to 46 SNPs and averaged 26 SNPs. The length of the LGs ranged from 29.9 cM to 89.2 cM and averaged 66.3 cM. The average inter-marker distance was 2.9 cM. The largest gap was located on LG09 with 23.6 cM (Table 3). An overview of the genetic map including marker density is displayed in Fig. 7.

**Table 3:** Summary of the genetic linkage map developed via regression mapping thinned to one marker per cM. The map comprises 259 SNP markers distributed across 10 linkage groups (LG).

Group	Number of SNPs	Length (cM)	Largest gap (cM)	Average gap (cM)
LG01	25	86.6	22.1	3.6
LG02	46	89.2	9.8	2
LG03	25	79.3	13.6	3.3
LG04	30	72	17.5	2.5
LG05	30	71.2	13.9	2.5
LG06	30	65.9	8.3	2.3
LG07	31	67	8.4	2.2
LG08	20	47.6	12.6	2.5
LG09	11	54.2	23.6	5.4
LG10	11	29.9	8.6	3
Average	25.9	66.3	-	2.9
Total	259	662.9	-	-

### QTL mapping (IM, MQM, Kruskal–Wallis), BLINK model & candidate genes

#### Flowering time and flowering type

**Fig. 8:**
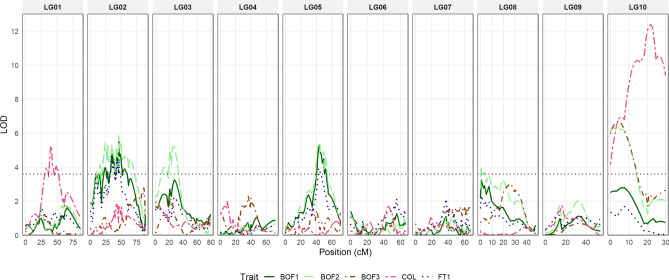
LOD curves rMQM. Results (LOD values) of rMQM for flowering time descriptors (BOF1 = darkgreen solid lines, BOF2 = lightgreen longdash lines, BOF3 = brown dashed-dotted line, FT1 = blue dotted lines) and flower color (COL = rose twodash lines). Dotted black line = Genome-wide significance threshold for BOF1, BOF3, FT1 and COL (LOD = 3.6), threshold for BOF2 (LOD = 3.5) not shown.

We conducted IM (Figure S2, raw results Table S1E) to identify candidate QTL regions followed by MQM (raw results in Table S1F and Table S1G for restricted MQM) to refine QTL positions and to detect potentially masked QTLs at subthreshold-maxima of IM. The genome wide LOD threshold (for α = 0.05) as calculated by the permutation test was 3.5 for BOF2 and 3.6 for BOF1, BOF3 and FT1, respectively. The final MQM for BOF2 revealed five significant QTLs on LG02, LG03, LG05, LG08 and LG10 (Fig. 8, Table 4). Interestingly, the QTL on LG05 had a subthreshold maximum in IM, but became a prominent QTL in MQM. Without mitigating strong difference between annuals and biennials (BOF1, FT1) only the QTLs on LG02 and LG05 surpassed the significance threshold (Fig. 8, Table S1H). In contrast, after removing the difference between annuals and biennials by separate Z-transformation (BOF3) the QTL on LG10 became most prominent (LOD = 6.73, explained variance = 14.7%, Fig. 8, Table S1H).

**Table 4:** Characteristics of significant QTL for flower color and flowering time (BOF2). Corresponding values for the IM and BLINK models are provided in parentheses () and square brackets [], respectively. 1-LOD intervals were extracted from the restricted MQM model. Expl. Var. indicates the percentage of explained phenotypic variance (%); Add. and Dom. represent additive and dominance effects in relation to the biennial allele, respectively. Significant scores (based on permutation tests for MQM/IM or FDR for BLINK) are indicated in bold.

**QTL**	**Trait descriptor**	**Group**	**Position** **cM**	**1-LOD** **cM**	**LOD** **MQM** **(IM)** **[BLINK]**	**Expl. Var** **%**	**Add** **MQM** **[BLINK]**	**Dom**	**Peak marker**
Cc-FT02.1	BOF2	LG02	45.9	41.9–51.2	**5.84** **(6.39)** **[7.47]**	9.2 [6.11]	13.17 [13.89]	2.66	contig_1125_12302196
Cc-FT03.1	BOF2	LG03	26.5	18.8–32.3	**5.23** **(3.14)** **[5.65]**	8.3 [4.18]	11.61 [11.17]	−3.36	contig_1045_5362953
Cc-FT05.1	BOF2	LG05	44.0	39.5–52.9	**5.39** (2.47) **[6.69]**	8.6 [4.82]	12.95 [12.68]	−2.53	contig_1514_1706860
Cc-FT08.1	BOF2	LG08	0.8	0.0–9.6	**3.93** (2.61) **[4.93]**	6.1 [2.84]	10.11 [10.03]	−0.26	contig_1688_9875227
Cc-FT10.1	BOF2	LG10	2.0	0.0–11.5	**6.32** **(5.61)** **[7.00]**	10.5 [6.90]	14.40 [13.05]	4.13	contig_880_18056394
Cc-COL01.1	COL	LG01	39.8	37.6–42.3	**5.25** **(5.33)** **[6.15]**	10.9	−0.49 [−0.5]	0.11	contig_460_5388281
Cc-COL10.1	COL	LG10	21.8	18.3–24.2	**12.39** **(13.11)** **[12.74]**	26.0	0.73 [0.7]	0.29	contig_1950_3018163

Results from classical QTL mapping were compared with results from a BLINK model (raw results in Table S1K). The FDR threshold of the BLINK model (for α = 0.05) was about 3.7. In general, BLINK corroborated the results of MQM (Table 4) with two deviations: Additionally, significant QTLs were detected for BOF1 on LG08 (as already detected for BOF2) as well as for FT1 on LG06 (Table S1K). Detected peak markers of BLINK are not identical but co-segregated with peak markers of MQM. The Kruskal Wallis test (Figure S3, Table S1L) for FT1 corroborated QTLs on LG02, LG03, LG05 and LG10, but also showed significant peaks (K* > 8) on LG06, LG07 and LG08. All significant QTLs from BLINK and the Kruskal–Wallis test not reaching the threshold in MQM are regarded as suggestive QTLs requiring additional evidence in subsequent studies. While following statistics focused on the five QTLs detected for BOF2, detailed statistics for the other trait descriptors and suggestive QTLs (including loci LOD > 2 from MQM, which might become of interest in future studies) are added to the supplements (Table S1H, Figure S4). Table S1H also includes an extended version of Table 4 for significant QTLs, providing 2-LOD intervals as well as flanking markers useful for fine mapping.

**Fig. 9:**
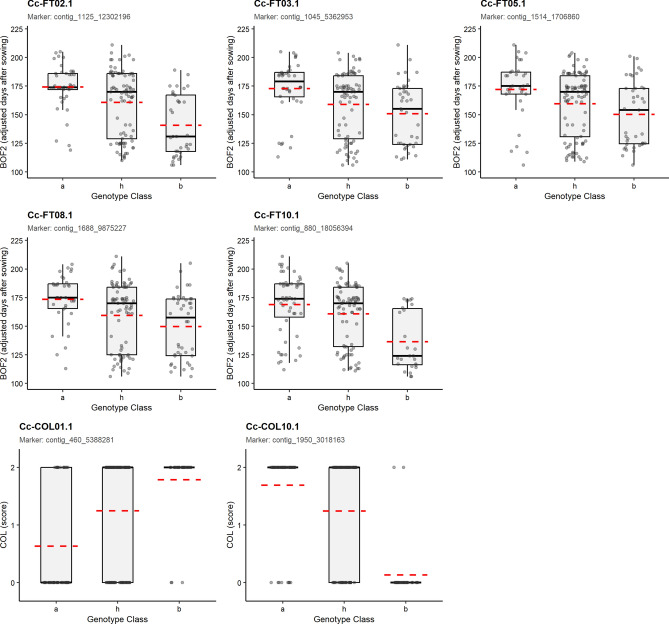
Allelic effects of identified QTL for flowering time (5, BOF2) and flower color (2, COL). Genotypes at peak markers: (a) homozygous biennial, (h) heterozygous, (b) homozygous annual. BOF2 is shown in adjusted days after sowing; COL is shown as scoring (0 = white, 2 = rose). Solid lines indicate medians; red dashed lines indicate means. Individual data points are shown as gray jitters.

**Fig. 10:**
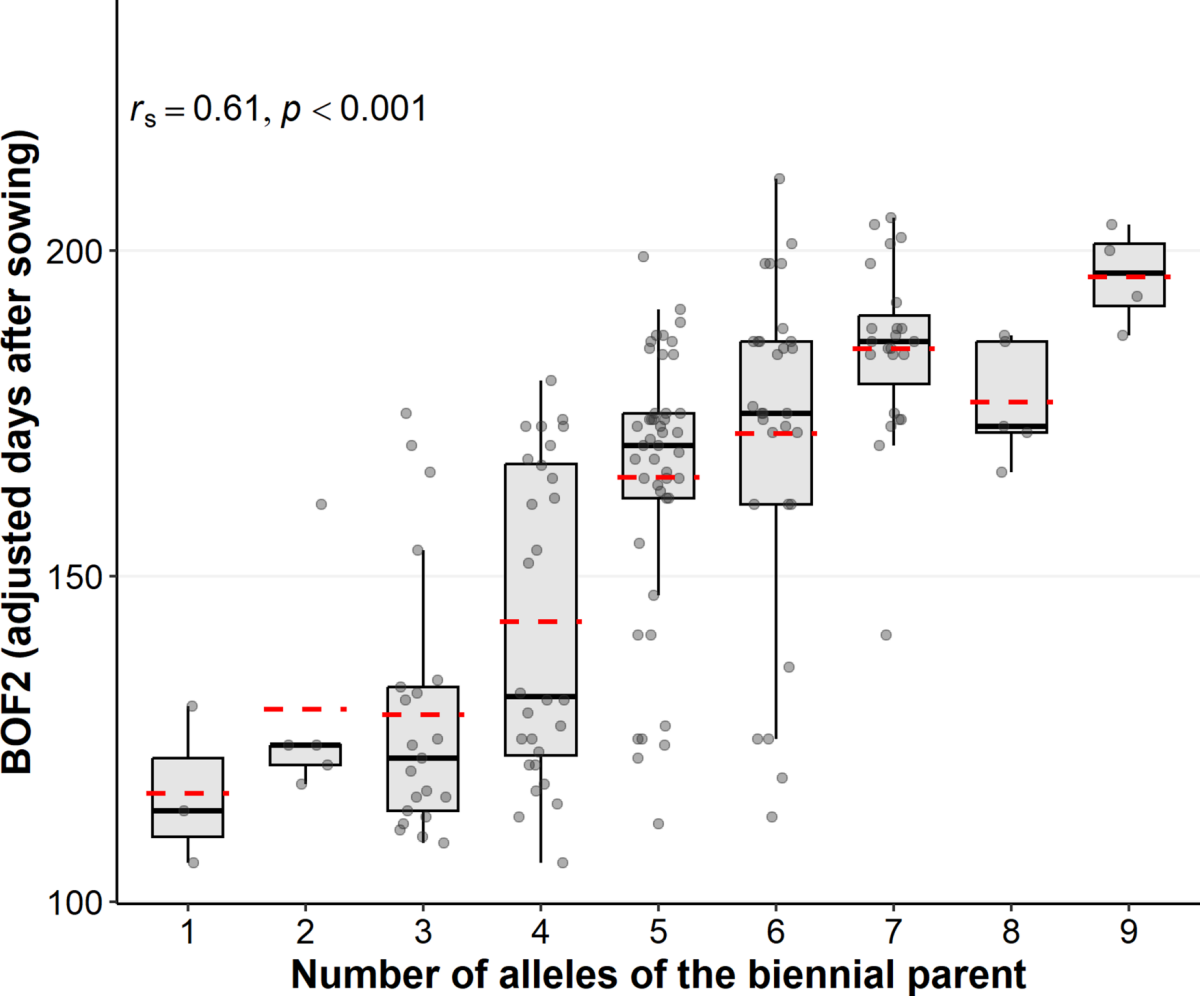
Phenotypic distribution of flowering time (BOF2). Based on the total number of biennial parent alleles across five identified QTL (Cc-FT02.1, Cc-FT03.1, Cc-FT05.1, Cc-FT08.1, Cc-FT10.1). Solid lines indicate medians; red dashed lines indicate means. Individual data points are shown as gray jitters.

The identified five QTLs for BOF2 explained 6.1% (Cc-FT08.1) to 10.5% (Cc-FT10.1) of the phenotypic variance, respectively (in total 42.7%). In relation to the biennial allele, these loci exhibited similar positive additive effects from 10.11 (Cc-FT08.1) to 14.40 (Cc-FT10.1) adjusted days after sowing. In comparison, dominance effects were low (−3.36 to 4.13 adjusted days after sowing; Table 4, Fig. 9). The 1-LOD interval ranged from 9.6 to 13.5 cM (Table 4). We found a medium correlation between the number of biennial alleles across the five QTLs and flowering time (BOF2, Spearman r = 0.61, Fig. 10). In detail, most plants with seven or more biennial alleles flowered in the second year (after 161 adjusted days after sowing), while plants with four to six biennial alleles exhibited high plasticity (Fig. 10). In addition, we scrutinized the effect of the QTLs on BOF2 within annuals and biennials, separately. Most conspicously, Cc-FT10.1 showed additive effects within both groups (Figure S5). Thus, Cc-FT10.1 contributed to flowering time in general but less to the strong difference between annuals and biennials, explaining why this QTL was masked in BOF1 without the adjustments in BOF2 or BOF3.

To identify potential regulators of flowering time, we exemplary screened for candidate genes within the 1-LOD intervals of Cc-FT02.1, which is most likely associated with vernalization requirement. We identified 1,571 genes (2,002 transcripts) within this interval (Table S4). Notably, a Histone-lysine N-methyltransferase (containing a SET domain and a RING/FYVE/PHD-type zinc finger) was identified in very close proximity to the peak of Cc-FT02.1 (32,536 bp; ~ 0 cM).

#### Flower color

**Fig. 11:**
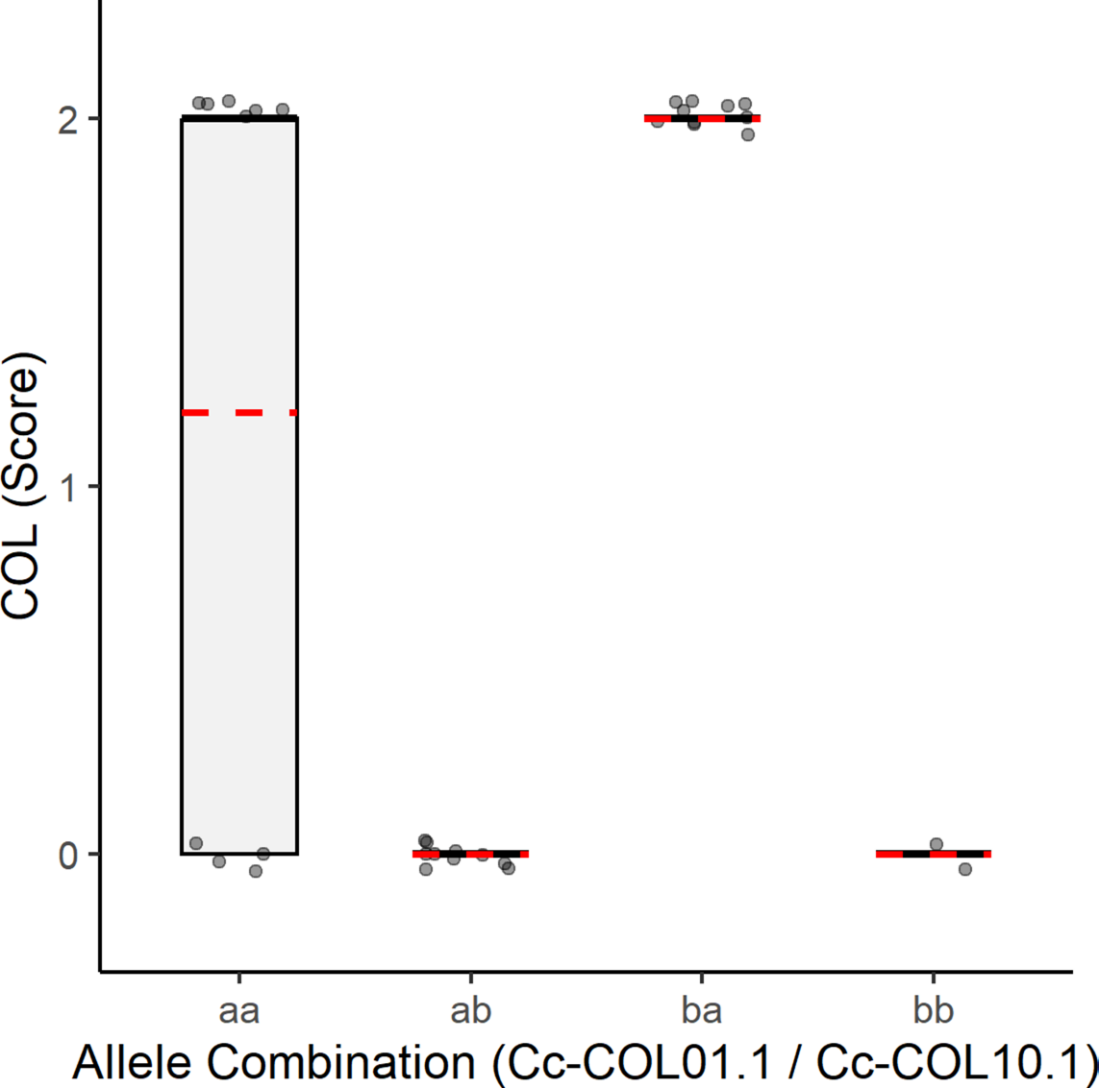
Phenotypic distribution of flower color for combinations of homozygous genotypes of two identified QTL (Cc-COL01.1 and Cc-COL10.1). Genotypes at peak markers: (a) homozygous biennial, (b) homozygous annual. COL is shown as scoring (0 = white, 2 = rose). Solid lines indicate medians; red dashed lines indicate means. Individual data points are shown as gray jitters.

The genome wide LOD threshold (for α = 0.05) as calculated by the permutation test was 3.6 for COL. Based on this threshold, initial interval mapping revealed two QTLs for COL on LG01 and LG10, which were corroborated by the final MQM, BLINK (Table 4, Fig. 9) and the Kruskal–Wallis test (Figure S3). At the major QTL on LG10 (explaining 26.0% of the variance, Table 4), most homozygous biennial (a) genotypes exhibited a rose flower, whereas most homozygous annual (b) genotypes exhibited a white flower (Fig. 9). Interestingly, the QTL on LG01 showed an opposing effect; specifically, most homozygous annual (b) genotypes showed a rose flower. For both QTLs, heterozygous genotypes were either white- or rose-flowering, with a slightly higher percentage of rose-flowering plants (Fig. 9). Analyzing the interaction of both QTLs at homozygous stages, we found that the biennial allele of Cc-COL10.1 was obligatory for rose flowering, but the annual allele at Cc-COL01.1 had a stabilizing effect (Fig. 11).

It must be noted as a statistical limitation that standard quantitative parameters, including additive effects, dominance effects, and explained phenotypic variance, were extracted from the parametric mixture models for the binary trait COL (Table 4, as also true for FT1, Table S1). Because these traits are nominal rather than continuously distributed, these values do not represent absolute quantitative physical metrics, but serve as mathematical approximations to indicate the relative genetic power and the phenotypic direction driven by the respective parental alleles.

We screened for candidate genes within the 1-LOD interval of Cc-COL10.1 and identified 320 genes (367 transcripts) within this interval (Table S4). Notably, three myb8 related transcription factors were located in a distance of 650,685 to 818,677 bp (approx. 1–2 cM) of the peak marker for Cc-COL10.1.

**Fig. 12:**
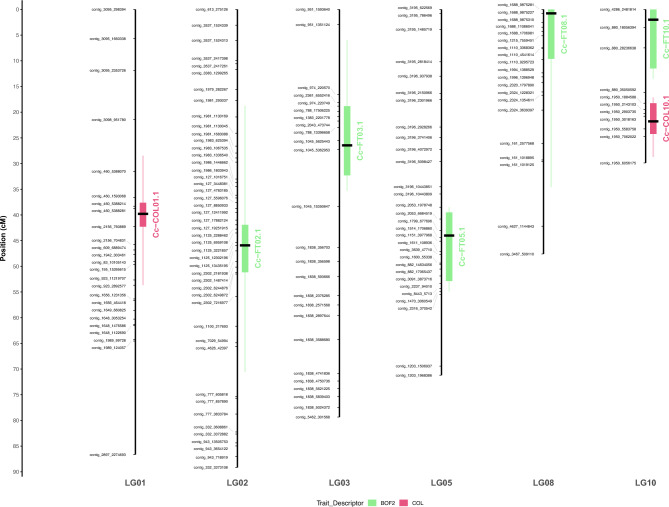
QTL positions on the genetic map (reduced to LGs covering a QTL). Black horizontal bars highlight the position of the peak from MQM. Thick and thin colored vertical bars show the 1-LOD and 2-LOD intervals, respectively.

Figure 12 summarizes the QTL positions on the genetic map for flower color and flowering time (BOF2).

## Discussion

### Annotated draft genome assembly and genetic map as research resource

The draft genome assembly presented here represents a significant milestone for caraway research, providing the first high-quality genomic backbone for this species. With a total size of 1.87 Gbp and a contig N50 of 9.5 Mb, the assembly demonstrates exceptional contiguity for a draft version. The high BUSCO completeness (97.9% for the genome, 98.6% for the annotation), combined with the low contamination rates, underscores the reliability of this resource. As a genetic resource, this assembly bridges the gap between traditional breeding and modern molecular genetics in caraway. As demonstrated in this study it allowed us to efficiently map GBS data compared to prior approaches using ‘mock references’^1^ and to identify candidate genes for detected QTLs. In future it may facilitate comparative genomics within the Apiaceae using the increasing number of available genomes in this family^23,37–40^ and support the development of tools for marker-assisted selection.

The caraway assembly (1.8 Gb) is smaller than the estimated cytometric genome size of 2.1 Gb. We attribute this ~ 14% difference to two main factors. First, cytometric genome-size estimates are relative measurements that are prone to overestimation due to the interference of cytosolic secondary metabolites with DNA-intercalating fluorochromes^41^. Second, high-copy repetitive sequences—such as centromeric and sub-telomeric stretches of satellite sequences, ribosomal DNA, and long, near-identical LTR-retrotransposons—cannot be fully resolved even with long reads; these regions tend to collapse during the assembly process, resulting in reduced copy numbers. Crucially, this underrepresented fraction occurs within the repeat space rather than the gene space, which is consistent with the 98.6% complete BUSCOs and the high read-mapping rate achieved here. For comparison, the barley MorexV3 pseudomolecules account for a total assembled genome size of 4.2 Gb, whereas its cytometric estimate was calculated at ~ 5 Gb, a gap equivalent to an entire chromosome that known missing repeats cannot fully explain^42^. An identical difference recurs across the 76 long-read genomes of the barley pangenome^43^. We therefore consider the present assembly a comprehensive representation of the caraway gene space, with the residual difference reflecting collapsed repeats and the upper-bound nature of the cytometric estimate.

Despite its high quality, the current assembly remains a draft at the scaffold level. While the contig N50 is high, the 2,171 fragments are not yet anchored into definitive chromosomes. Here, the given genetic map offers first possibilities to resolve the physical orientation of the scaffolds, but a high number of scaffolds is not represented by SNPs. Future efforts should employ Hi-C sequencing^44^ to bridge the remaining gaps and achieve a true chromosome-level assembly. While structural annotation is excellent, transcriptomics (RNA-Seq) could further refine the gene models, particularly for isoforms and non-coding RNAs.

The wild biennial parent utilized in this study exhibited a high baseline heterozygosity (24.81%, Table CCC), which could be expected for a predominant outbreeding species^45^. Including these heterozygous loci would have increased the number of SNPs for mapping. However, this would have introduced structurally divergent segregation patterns (resembling a pseudo-testcross) and potentially GBS-related technical artifacts like pseudo-heterozygosity from paralogous regions^46^. Indeed, this is supported by the fact that the annual inbred parent of the fifth inbred generation retained an observed heterozygosity of 5.92%, which likely overestimates true biological heterozygosity due to such technical noise. To prevent severe mapping errors in the absence of a chromosome-level reference map for validation, we deliberately chose to exclude parental heterozygous loci. In general, we applied strict, conservative filter settings and focused on a lower but sufficient number of homozygous loci to construct a first robust map.

### Flowering time and flowering type

#### Genetic architecture of flowering time and flowering type in caraway – beyond the 3:1 mendelian ratio

In the following discussion it is crucial to differentiate between flowering time as a quantitative trait and flowering type (annual vs. biennial) as a qualitative trait. In this study, ‘flowering time’ usually included the prevalent quantitative difference between flowering types (BOF1 and BOF2) so that we predominately searched for loci controlling flowering type using these trait descriptors. However, we also searched for flowering time loci independent from flowering type using Z-standardization (BOF3). While flowering type is primarily associated with vernalization requirement in caraway^3^, other mechanisms may contribute, such as strong general flowering time regulators shifting flowering induction beyond the first vegetation period. Therefore, it can become challenging to disentangle general flowering time loci from specific loci governing vernalization response.

We will focus on the five-QTL model supported by the final MQM model and corroborated by BLINK for BOF2 using adjusted flowering time. The mitigation of the bimodal distribution of data (compared to BOF1 and FT1) clearly improved the signal for QTL detection, both for loci rather related to vernalization response and to flowering time in general (see below). Additional loci detected only by Kruskal–Wallis (for FT1) or BLINK as well as sub-threshold signals in MQM might represent minor-effect QTL and should be interpreted cautiously. Overall, the integration of MQM, IM, BLINK, and Kruskal–Wallis analyses (for nominal scaled data) allowed us to distinguish high-confidence QTLs from suggestive loci, providing a conservative yet comprehensive characterization of the genetic architecture of flowering traits in this F_2_ population. To emphasize the explorative character of this study, all suggestive loci are presented in the supplements. As a side note, BLINK although designed for GWAS, proved to be a fast and efficient tool to detect QTLs in F_2_ populations. However, without a chromosome-level assembly, this model cannot replace the complex mapping pipeline.

Our observations in F_1_ generation confirm a dominant annual flowering type when assessed as a qualitative trait, which initially aligns with earlier literature^3,6^. However, when treated as a quantitative trait, we observed a considerable delay in flowering time in F_1_ plants compared to annual parents. This suggests that while ‘annuality’ per se may appear dominant (qualitative view), the underlying flowering time alleles exhibit incomplete dominance or a strong additive component (quantitative view).

This was further corroborated by the F_2_ field trial: While all plants of the annual parent reached anthesis, only a few F_2_ plants flowered within the same timeframe. The observed higher proportion of biennials in F_2_ can only be explained on a quantitative scale in combination with the late sowing date, which may have masked the ‘annual’ phenotype of late-flowering genotypes. This biological truncation further justifies the use of the adjusted BOF2 trait descriptor for flowering time, which better reflects the true genetic potential of these individuals. Indeed, the five detected QTLs for BOF2 predominantly showed additive effects with only marginal dominance. No QTL showed an obligatory biennial habit for the homozygous biennial genotype (Fig. 9), so that it is unlikely that a single vernalization switch operates in this specific population. This supports the hypothesis that flowering time in this cross is not governed by a single 'on/off’ switch, but by several minor-to-medium effect QTL (6.1 to 10.5% explained variance, Table 4). Therefore, our data do not support a major QTL affecting flowering type with a classic 3:1 ratio (annual vs. biennial) associated with vernalization requirement as proposed by Nemeth and Pluhar^6^. This discrepancy might stem from the distinct genetic background of our Russian wild biennial parent compared to the cultivar ‘Maud’ used by Nemeth and Pluhar^6^.

Nevertheless, since flowering of biennials can be induced by cold, the vernalization pathway must play a crucial role in flowering time regulation in caraway. Although we cannot fully disentangle whether the detected QTLs modulate vernalization requirement or flowering time in general or both, comparing the result of the different flowering time descriptors (BOF1, BOF2, BOF3 and FT1) and the effects within flowering types can provide first hints, which, however, need additional evidence: Since, the effect of the QTLs Cc-FT02.1, Cc-FT03.1, Cc-FT05.1, and Cc-FT08.1 is predominantly explained by the differentiation between annuals and biennials (no significance for BOF3) these QTLs are likely involved in the vernalization pathway. In contrast, QTL Cc-FT10.1 exhibited considerable effects within both flowering types (high significance for BOF3). This suggests that Cc-FT10.1 acts as a general flowering time regulator, potentially explaining why the Russian wild genotype shows delayed flowering even among other biennial genotypes^7^. As a simplified working thesis, we finally propose a polygenic model where Cc-FT02.1, Cc-FT03.1, Cc-FT05.1, and Cc-FT08.1 primarily but not exclusively modulate vernalization response, whereas Cc-FT10.1 acts as a general flowering time regulator.

#### Comparison to other Apiaceae and candidate genes

In Apiaceae vegetables and medicinal plants grown for their root structures, early/annual flowering (or bolting) has been analyzed as detrimental behavior reducing quality and yield^47,48^, whereas genetic studies on flowering time in seed drugs seem to be scarce. Similar to caraway, in multiple Apiaceae species annual flowering habit was found to be dominant. In carrot one vernalization locus has been mapped to chromosome 2^49^, yet the monogenic model was later extended to a multiallelic digenic model^50^. In celery one major locus was identified for controlling vernalization requirement^51^. On first glance, these studies appear to contrast with our proposed polygenic model for the investigated F_2_ in caraway. However, it should be recognized that each study merely represents a snapshot of the genetic diversity within a species or family. Looking into details, we see difficulties to distinguish between a qualitative (“annual”) and quantitative (“early”) description of flowering in other species, too. E.g. in carrot even annuals need a cold stimulus^52^. Moreover, various reports of different rates of early/annual flowering in diverse Apiaceae species depending on environmental conditions and cultivation systems, including sowing date^48^, demonstrate some complexity of the genetic control of flowering in Apiaceae.

The transition from vegetative to reproductive growth in plants is governed by a complex integrated network of pathways. While transcriptomic studies in *Angelica sinensis* and *Daucus carota* have identified a wide array of orthologs^53–55^, pinning down genes behind certain flowering time QTLs remains challenging even in well-studied Apiaceae like carrot. Genes involved in the cold-induced suppression of floral repressors (e.g., *FLC*-like) or the activation of vernalization response genes (e.g., *VRN*-like) represent the most promising candidates for QTLs predominantly associated with flowering type, such as Cc-FT02.1.

Indeed, we identified a Histone-lysine N-methyltransferase (containing a SET domain and a RING/FYVE/PHD-type zinc finger) in close proximity to the peak marker of Cc-FT02.1. Based on its predicted domain architecture, this gene represents a plausible candidate for involvement in chromatin-mediated vernalization responses. In particular, the combination of SET and PHD domains is reminiscent of functions associated with Polycomb-mediated regulation and cold-responsive chromatin remodeling described for vernalization-related factors such as *VRN2* or *VIN3*^56,57^. However, this functional relationship remains hypothetical and cannot be inferred from positional information alone. The inherent uncertainty of the peak position and the high gene density within the 1-LOD interval must be considered, especially since this interval may not represent a conservative confidence threshold in a medium-sized F_2_ population. Therefore, fine-mapping and subsequent functional validation remain indispensable to confirm the causal genes underlying Cc-FT02.1 and other identified QTLs.

### Flower color

#### Genetic architecture of flower color – environmental sensitivity, an expected major “biennial” QTL & a surprising modifying ‘annual’ QTL

Whereas the biennial parent showed a deep rose flowering habit in our field trials in 2019 and 2020^7^, the substantial bleaching of flower color in both the parent and the F_1_ generation under greenhouse conditions indicated that flower color in caraway is a plastic trait, highly sensitive to environmental factors. Observations from various field trials further show that most caraway genotypes, including annuals, can produce rose/reddish pigments under stress conditions (though not reaching the deep rose intensity shown in Fig. 1), which adds an additional layer of complexity to phenotyping.

Under field conditions, the rose coloration reappeared in the F_2_ generation with varying intensities; however, environmental effects (bleaching) were again confirmed on sunny days. Since a quantitative scoring of color intensity was considered insufficiently robust and reproducible due to these environmental effects, in this study we focused on presence and absence of flower coloration. Disregarding color intensity, the major QTL detected on LG10 showed incomplete dominance of the biennial rose-flowering allele (Table 4). Given that flower color could not be assessed on a quantitative scale and that heterozygotes exhibited high plasticity (Fig. 9), this might suggest an intermediate inheritance pattern.

In addition to the major locus on LG10, a second QTL was identified on LG01. Interestingly, the annual allele at this locus contributed positively to rose coloration. It might be that we observe an epistatic effect between both QTLs in the F_2_ population that was latent in the parental lines, as the combination of homozygous annual alleles on LG01 and homozygous biennial alleles on LG10 resulted in a consistent rose phenotype (Fig. 11). This indicates that while LG10 may act as a primary switch, the LG01 locus functions as a genetic modifier. Furthermore, the observation that this pigmentation extends beyond the petals to the internodes, peduncles, and ripening seeds suggests a systemic regulation of pigment biosynthesis rather than an organ-specific floral trait.

#### Comparison to other Apiaceae and candidate genes

Likely, flower color is associated with anthocyanin synthesis. Purple carrots, which “accumulate large quantities of anthocyanins in their root tissues, as well as in other plant parts”^58^ might be the best characterized model within Apiaceae. Here, two QTLs (P_1_ and P_3_) have been mapped to chromosome 3^59,60^. It has further been shown that the MYB transcription factor DcMYB7 (within P_3_), rather than structural genes, controls purple pigmentation in carrot roots^58,61^. In caraway, it is plausible that similar to carrot rather regulatory genes may be responsible for the difference between rose- and white-flowering genotypes. The fact that white-flowering genotypes can develop rose pigmentation under stress conditions (based on own observations) is consistent with the hypothesis that the structural biosynthetic pathway is intact and only lacks a regulatory trigger. Therefore, the detected three myb8 related transcription factors nearby the peak for Cc-COL10.1 represent plausible candidate genes. However, again fine-mapping and subsequent functional validation remain indispensable to confirm the causal genes.

## Limitations and future perspectives

In general, all findings of QTL mapping are restricted to the evaluated biparental population and one specific environment. A validation across different populations and environments remains necessary. Without such a validation, all detected QTLs should be considered preliminary. Moreover, we used a moderate sample size (N = up to 187) for QTL mapping, which limits the detection of small-effect QTLs. Therefore, considering the polygenic nature of flowering induction observed in our population, in future validations the sample size should be increased. Although we detected plausible candidate genes, at the current state these findings are highly speculative. QTL confidence intervals are still very large and include an extensive list of annotated genes. Further studies, such as fine-mapping and gene expression analysis are necessary to narrow down QTL intervals and the list of potential candidate genes.

For future research on flowering time, the inclusion of cultivated biennial material could be of particular interest. This would also allow to validate the observations of Nemeth and Pluhar^6^, who proposed one major QTL affecting flowering time and flowering type in the cultivar ‘Maud’. Since we found that a relatively late sowing potentially masked the “true” flowering type of F_2_ plants, it seems recommendable to use earlier sowing dates in subsequent studies.

Although the environmental sensitivity of flower color in caraway complicates its practical application, it remains a trait of interest for the development of biennial or winter-annual cultivars. Such varieties, which typically flower under milder spring conditions, are more likely to consistently display the rose phenotype, offering opportunities for specialized branding and market differentiation. Beyond agriculture, the aesthetic combination of rose flowers, subtly tinted seeds, and pigmented stalks suggests a potential for ornamental or floristic use.

## Conclusion

The QTL mapping in this study provides novel insights into the genetic architecture of flowering time and flower color in caraway establishing a crucial foundation for future functional studies. Together, the linkage map and the annotated draft genome assembly represent a milestone resource for caraway, which will promote the molecular elucidation of important traits that have remained uncharacterized until now.

## Supplementary Information

Supplementary Information 1.

Supplementary Information 2.

Supplementary Information 3.

Supplementary Information 4.

Supplementary Information 5.

Supplementary Information 6.

Supplementary Information 7.

Supplementary Information 8.

Supplementary Information 9.

## Data Availability

Phenotypic data, filtering pipeline, SNP matrix, genetic maps, results from QTL mapping, functional annotation and list of candidate genes are added to the supplements. The GBS data (FASTQ files) generated for this study have been deposited in the European Nucleotide Archive (ENA) under the project accession number PRJEB108909. Individual sample accessions are listed in Supplementary Table S1M. The long-read whole genome sequencing data collected in this study, the genome assembly and annotation have been deposited at the European Nucleotide Archive (ENA) under the project accession number PRJEB111921.
