# Supplementary figures and images for: Dissecting flowering time and flower color in Carum carvi utilizing a long-read draft genome and a GBS-based QTL mapping
Source: Sci Rep. 2026 Jul 14;16:22067. doi: 10.1038/s41598-026-61767-1 (PMC13370025; doi:10.1038/s41598-026-61767-1)

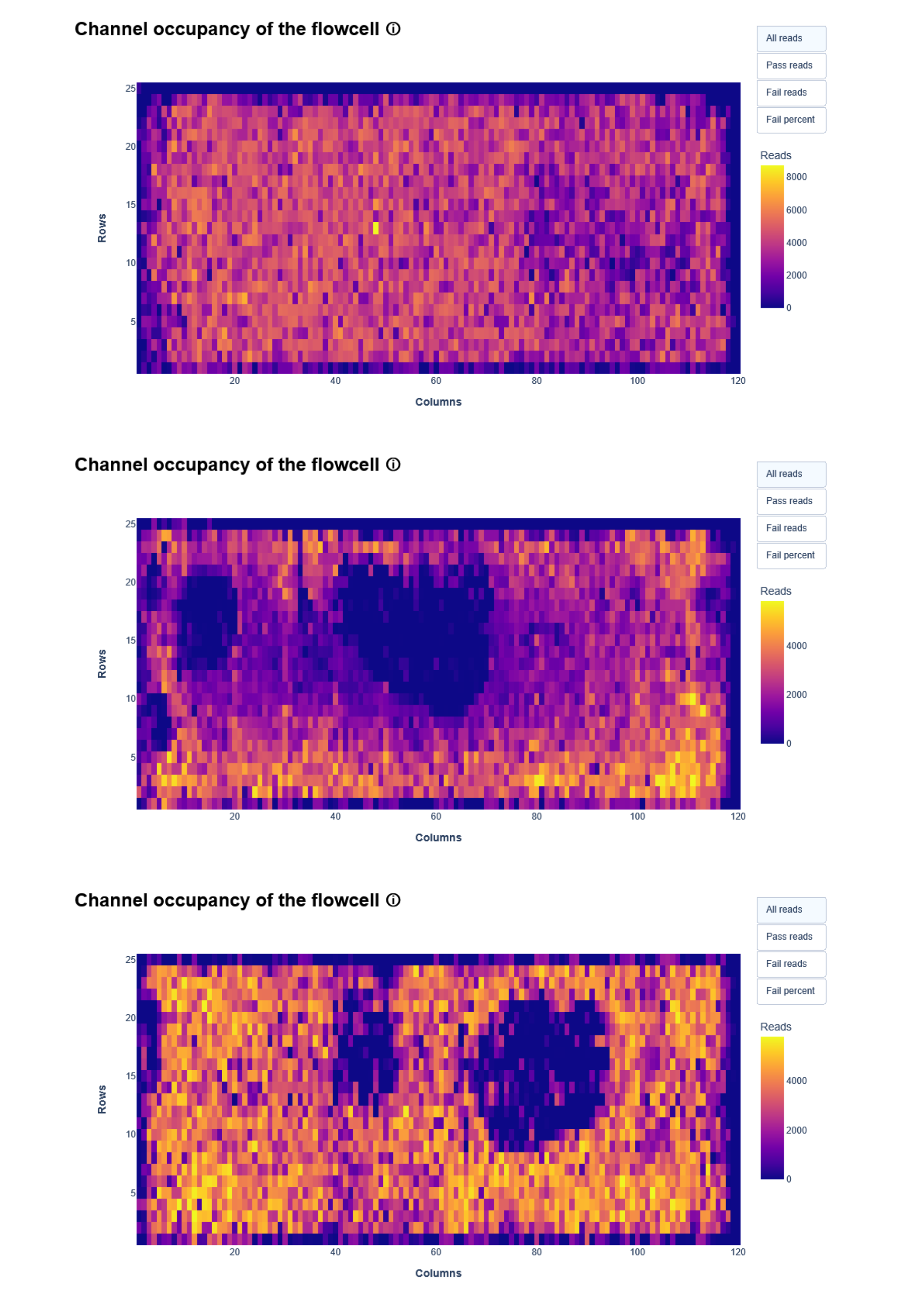

Supplement: Supplementary file 1 — Supplementary Information 1. [file 41598_2026_61767_MOESM1_ESM.tiff]

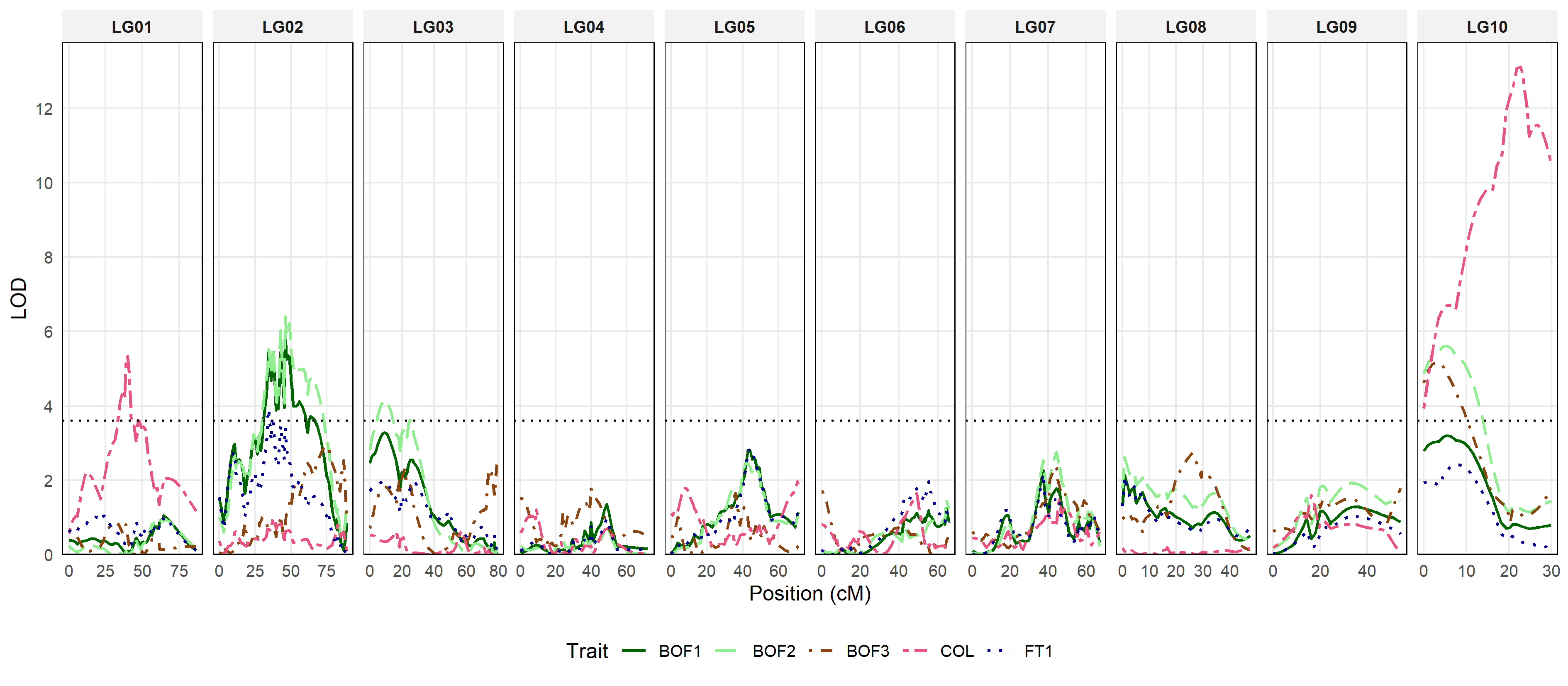

Supplement: Supplementary file 2 — Supplementary Information 2. [file 41598_2026_61767_MOESM2_ESM.tiff]

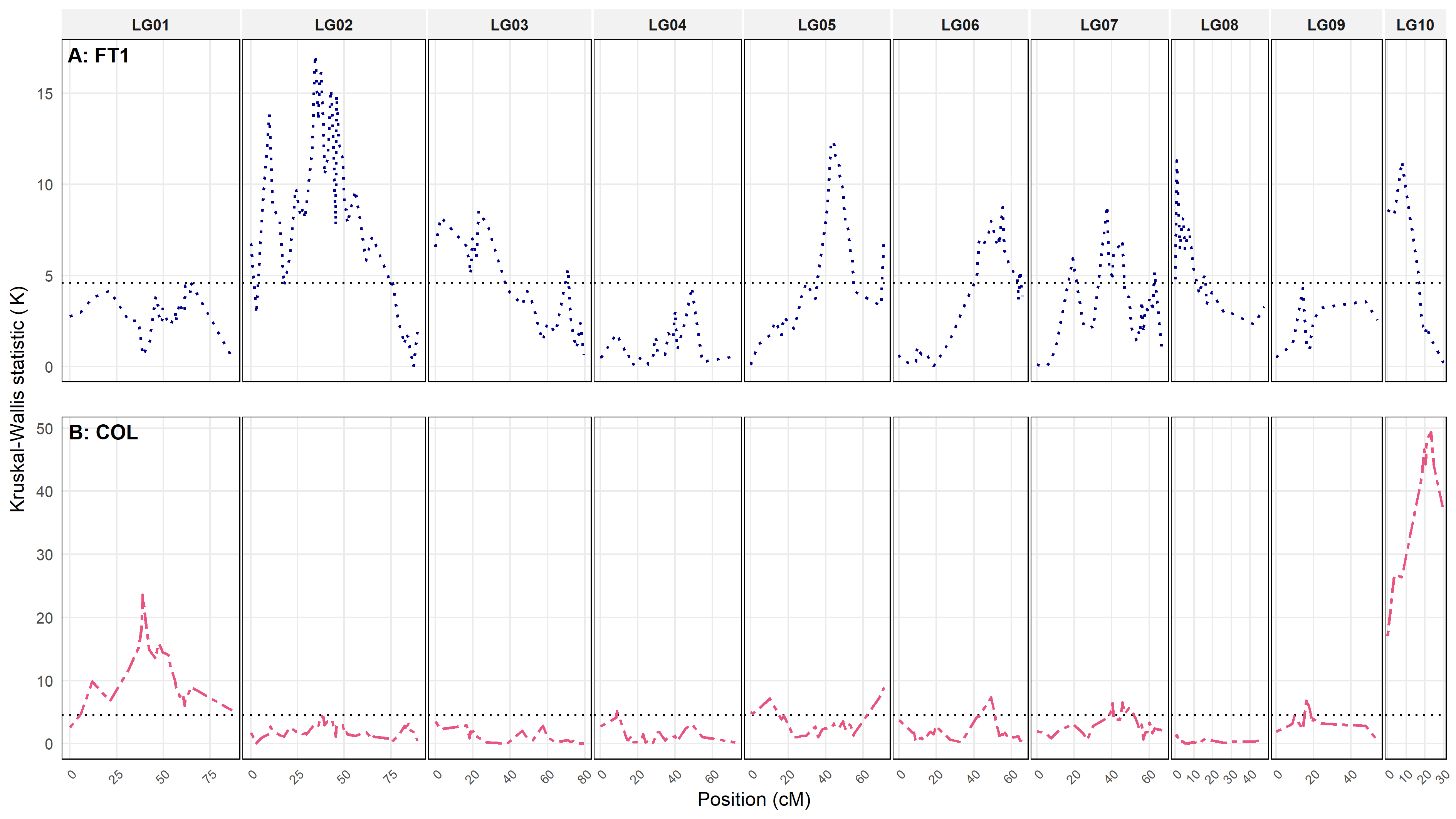

Supplement: Supplementary file 3 — Supplementary Information 3. [file 41598_2026_61767_MOESM3_ESM.tiff]

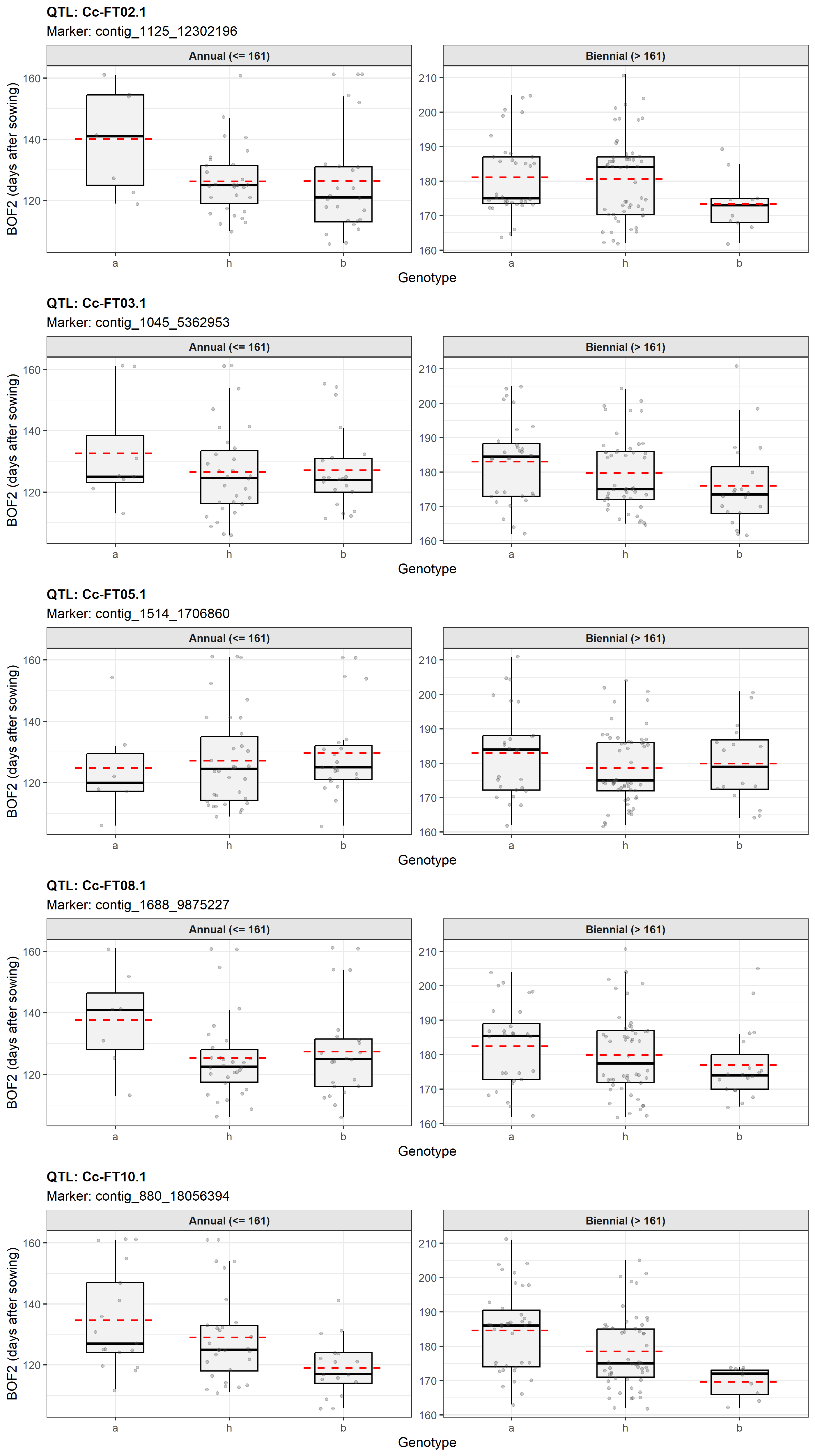

Supplement: Supplementary file 5 — Supplementary Information 5. [file 41598_2026_61767_MOESM5_ESM.tiff]
