# Supplementary material for: Dissecting flowering time and flower color in Carum carvi utilizing a long-read draft genome and a GBS-based QTL mapping
Source: Sci Rep. 2026 Jul 14;16:22067. doi: 10.1038/s41598-026-61767-1 (PMC13370025; doi:10.1038/s41598-026-61767-1)
Supplement: Supplementary file 4 — Supplementary Information 4. [file 41598_2026_61767_MOESM4_ESM.pdf]

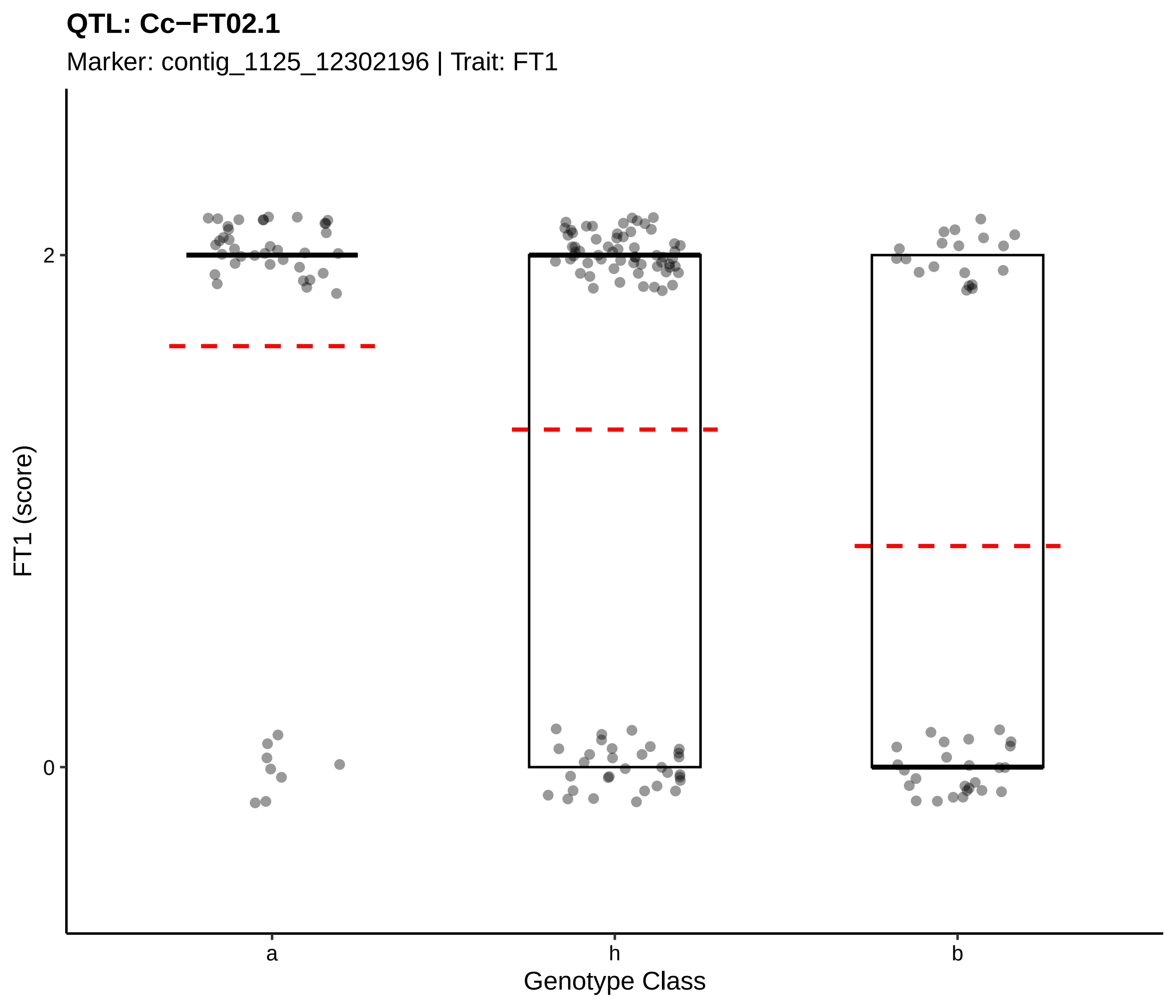
QTL: Cc−FT02.1
Marker: contig_1125_12302196 | Trait: FT1
2
0
FT1 (score)
a
h
b
Genotype Class

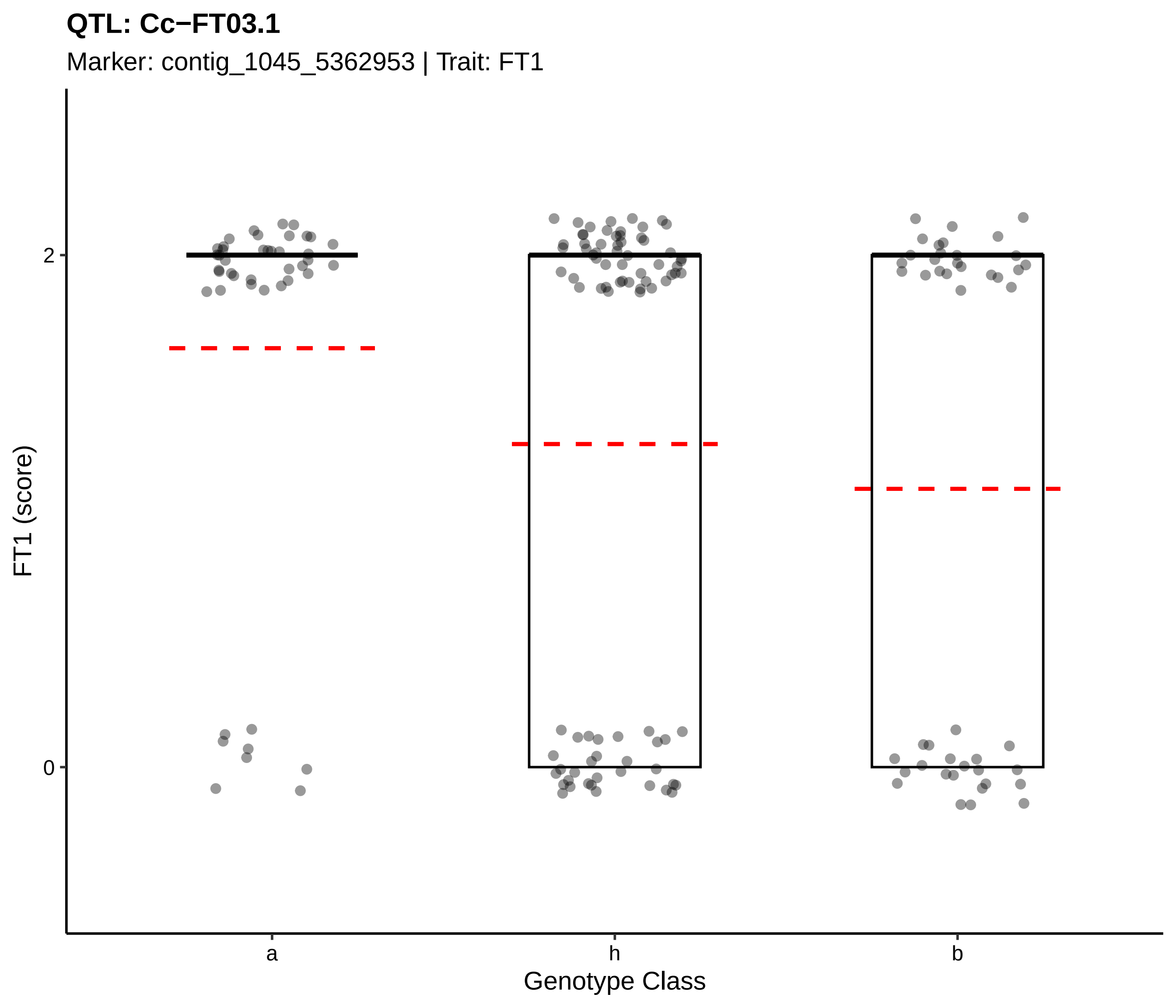

QTL: Cc−FT03.1
Marker: contig_1045_5362953 | Trait: FT1
FT1 (score)
2
0
a
h
b
Genotype Class

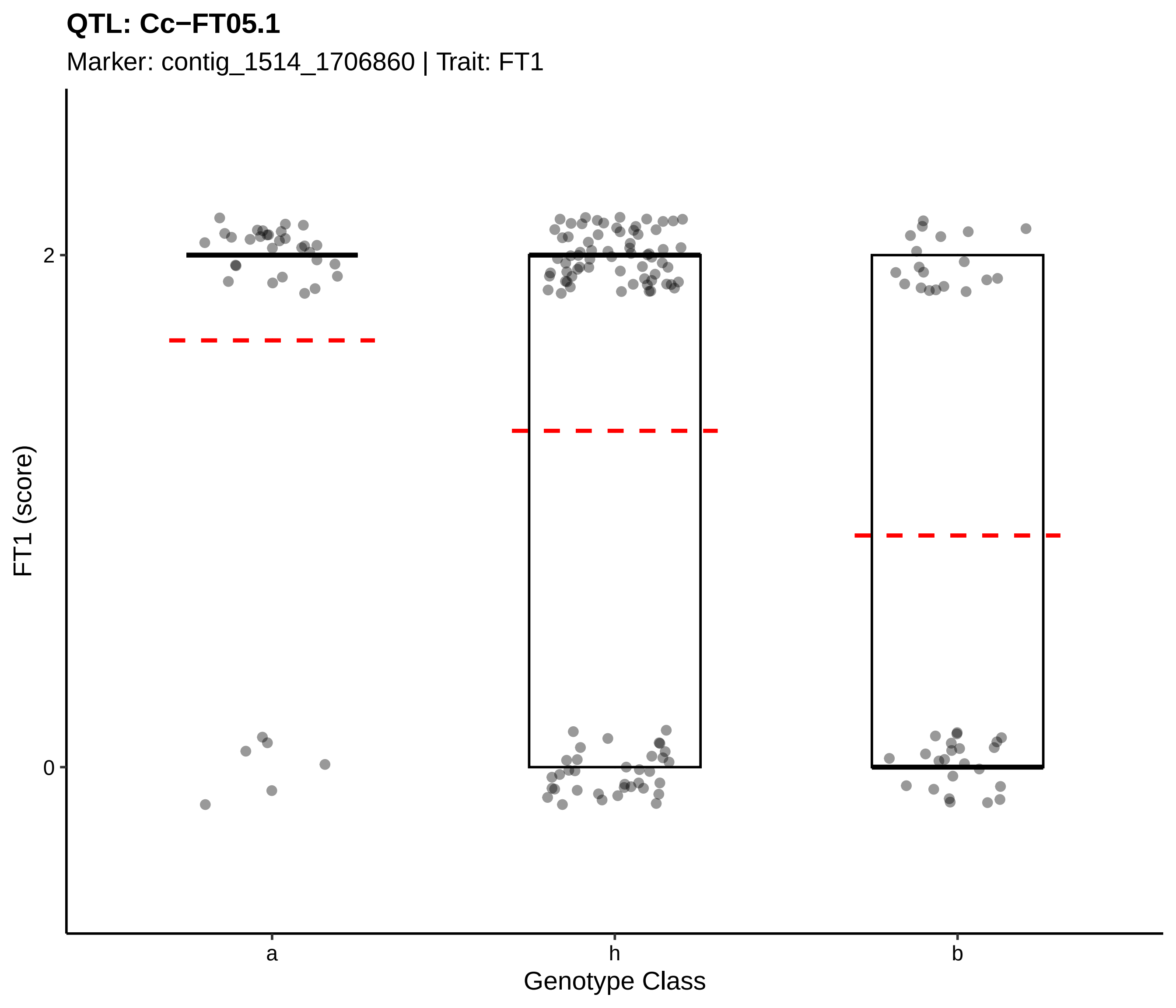
QTL: Cc−FT05.1
Marker: contig_1514_1706860 | Trait: FT1
FT1 (score)
2
0
a
h
b
Genotype Class

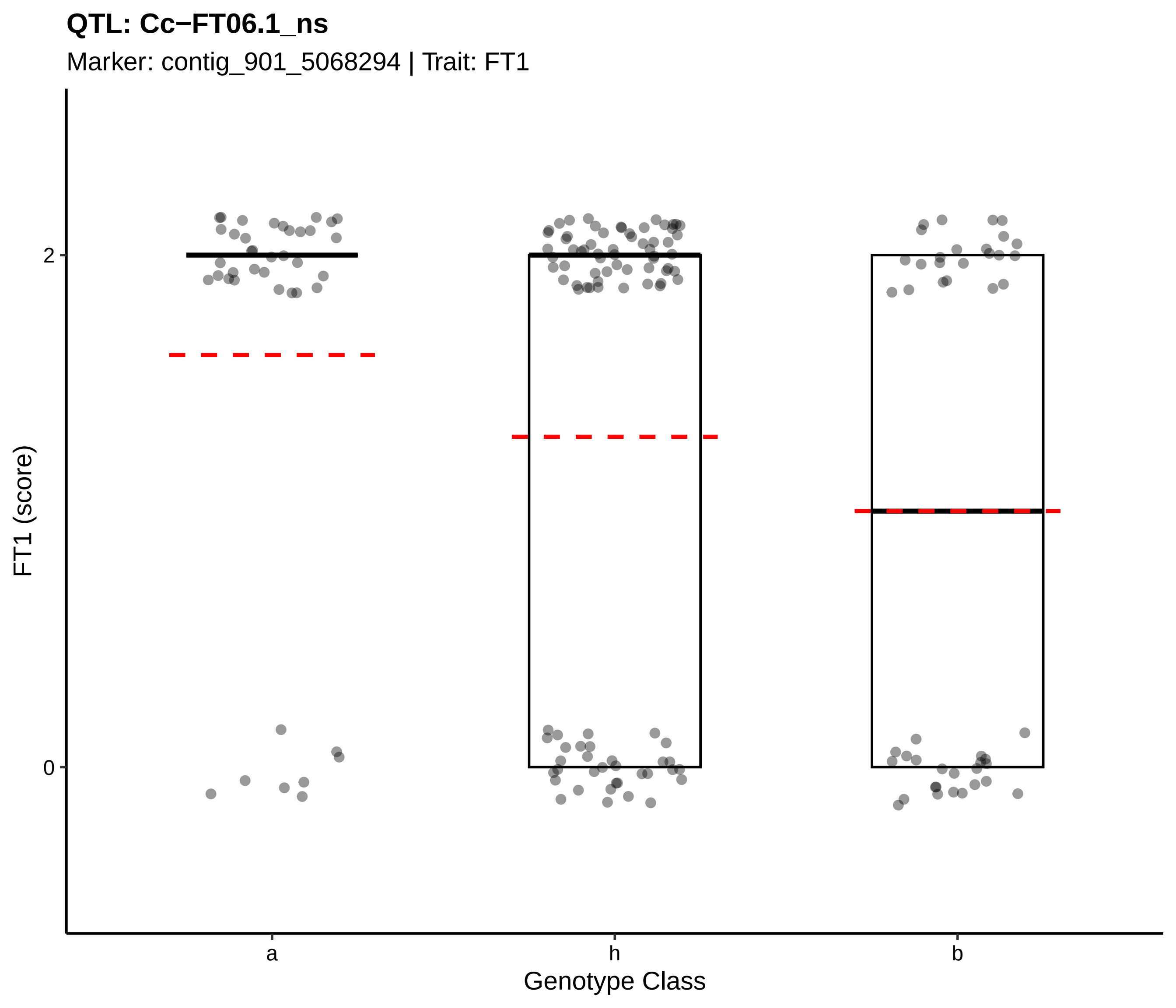
QTL: Cc−FT06.1_ns
Marker: contig_901_5068294 | Trait: FT1
FT1 (score)
2
0
a
h
b
Genotype Class

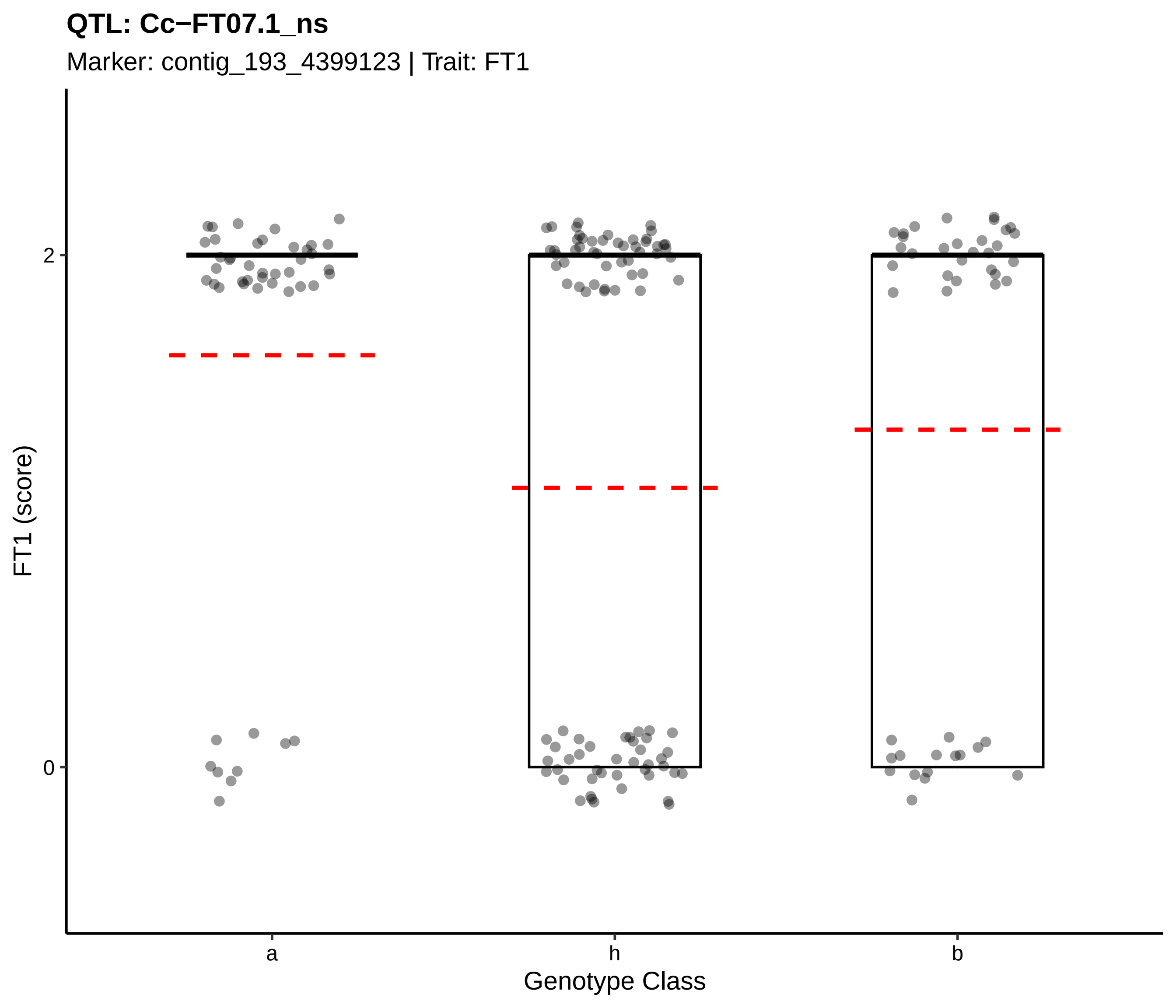

QTL: Cc−FT07.1_ns
Marker: contig_193_4399123 | Trait: FT1
FT1 (score)
2
0
a
h
b
Genotype Class

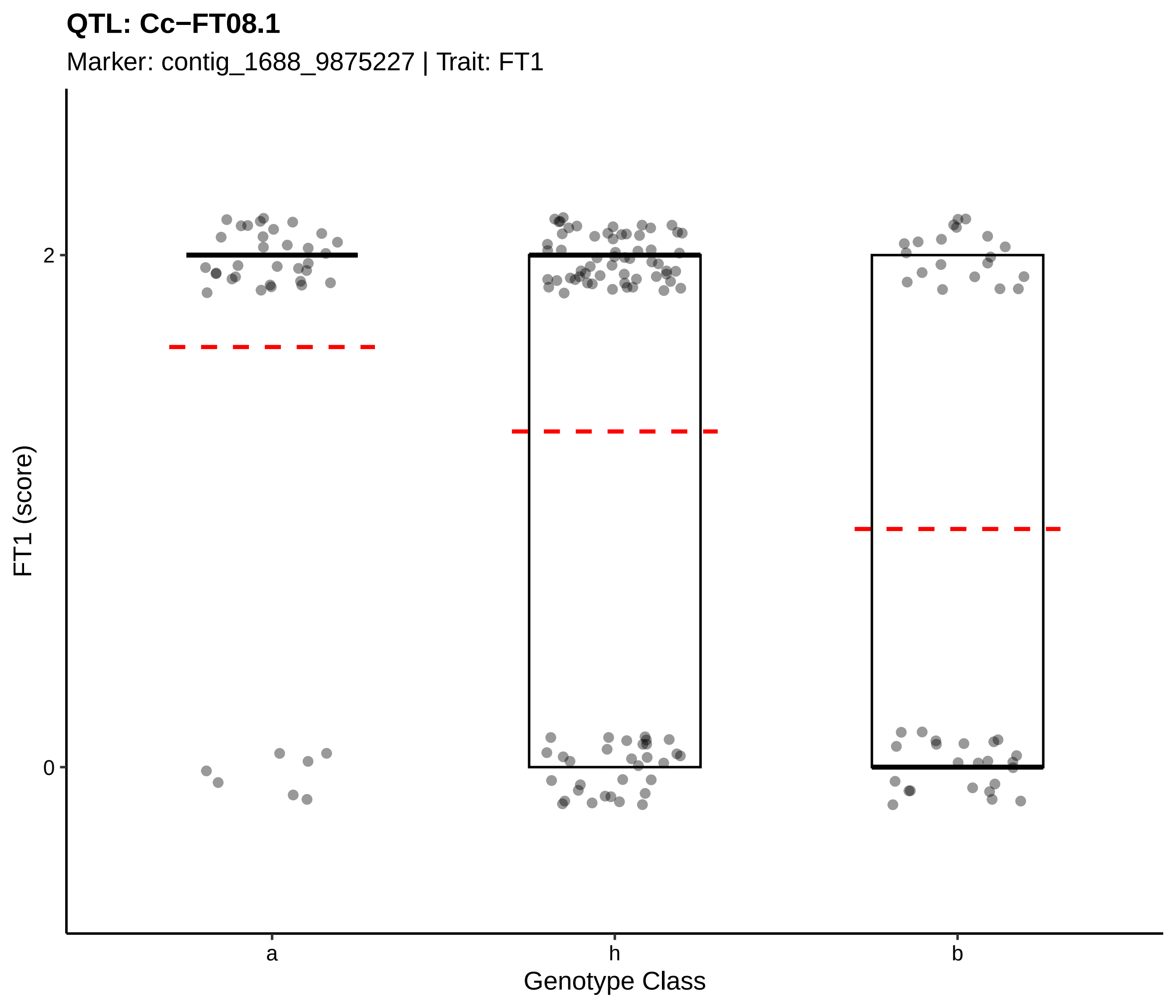

QTL: Cc−FT08.1
Marker: contig_1688_9875227 | Trait: FT1
FT1 (score)
2
0
a
h
b
Genotype Class

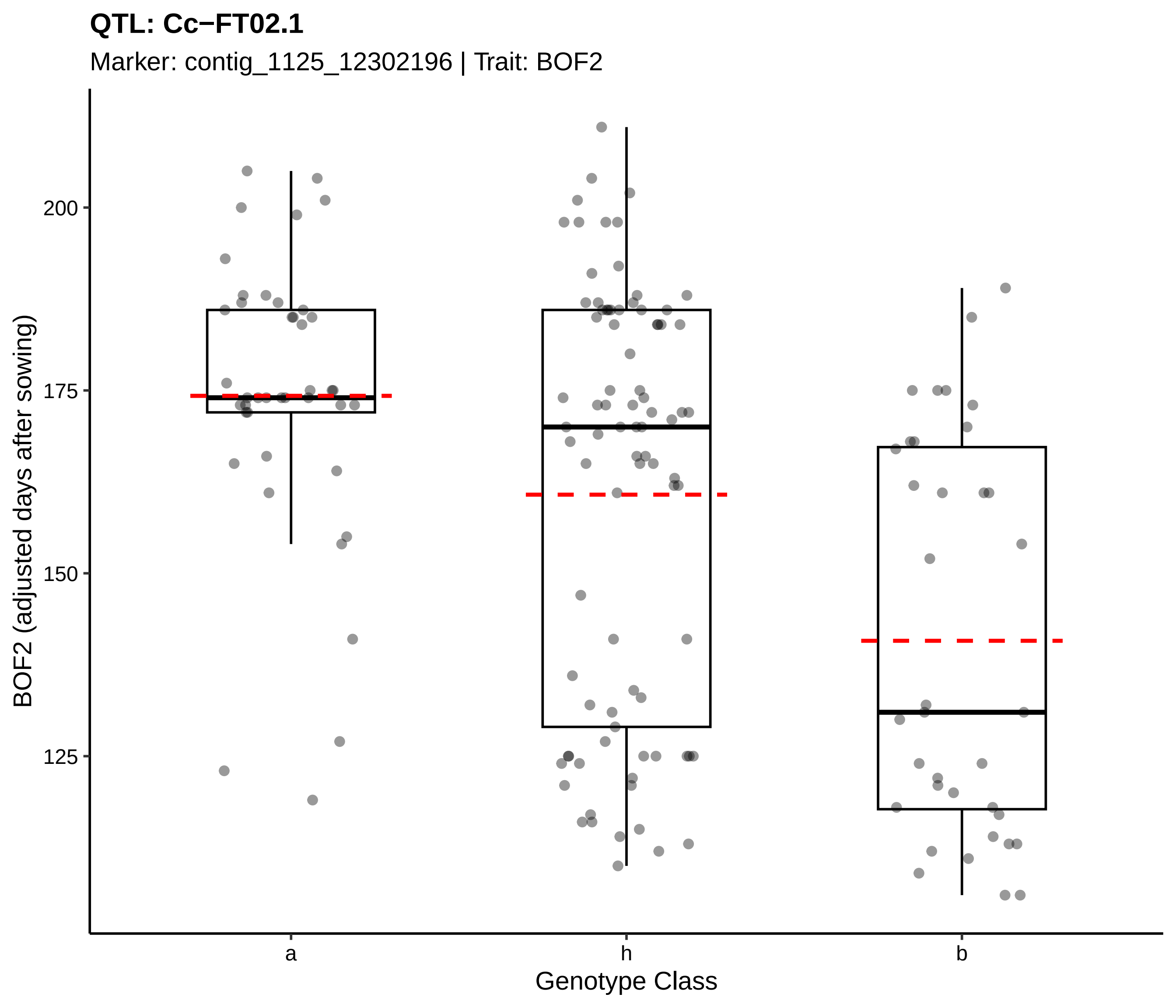

QTL: Cc−FT02.1
Marker: contig_1125_12302196 | Trait: BOF2
BOF2 (adjusted days after sowing)
200
175
150
125
a
h
b
Genotype Class

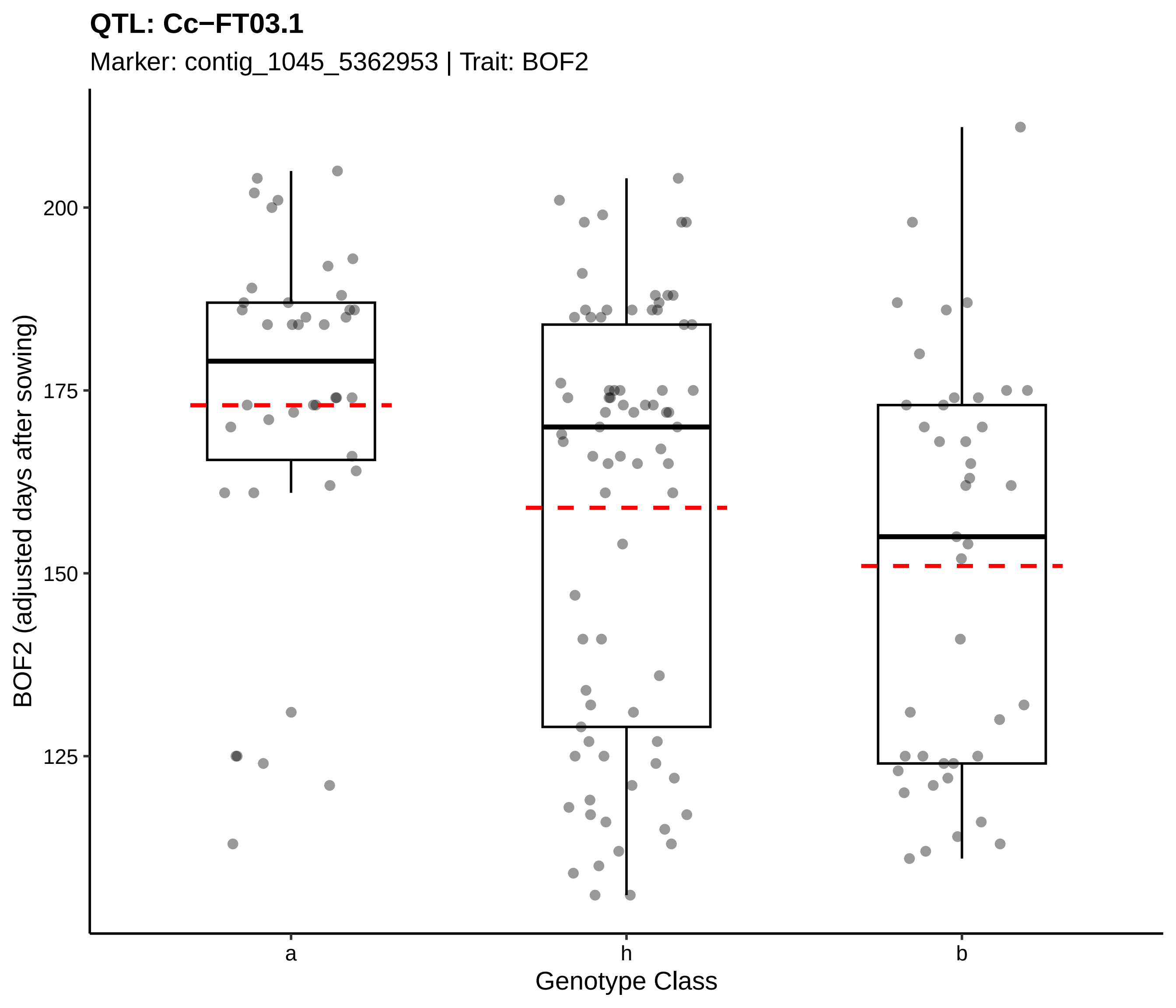
QTL: Cc−FT03.1
Marker: contig_1045_5362953 | Trait: BOF2
BOF2 (adjusted days after sowing)
200
175
150
125
a
h
b
Genotype Class

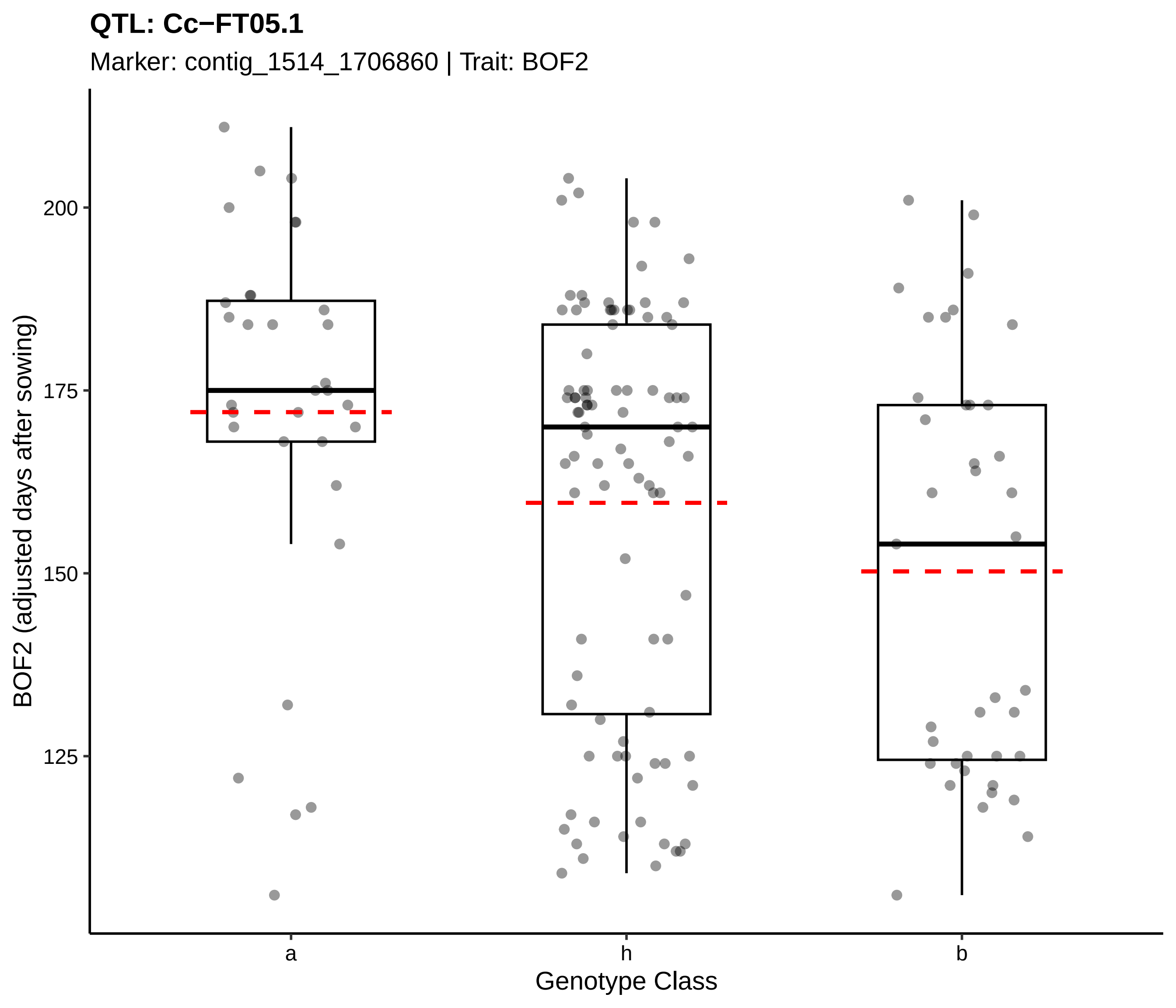
QTL: Cc−FT05.1
Marker: contig_1514_1706860 | Trait: BOF2
BOF2 (adjusted days after sowing)
200
175
150
125
a
h
b
Genotype Class

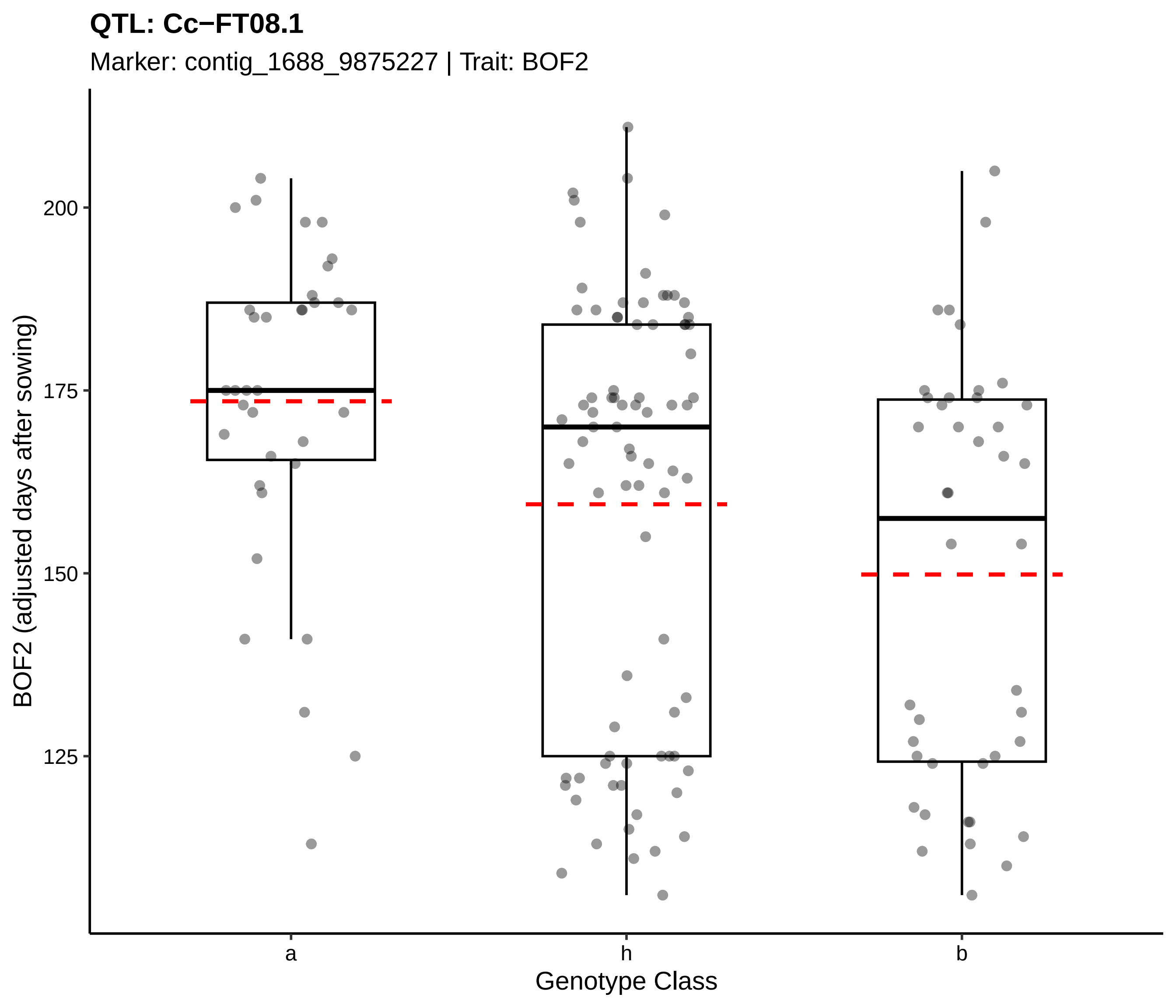
QTL: Cc−FT08.1
Marker: contig_1688_9875227 | Trait: BOF2
BOF2 (adjusted days after sowing)
200
175
150
125
a
h
b
Genotype Class

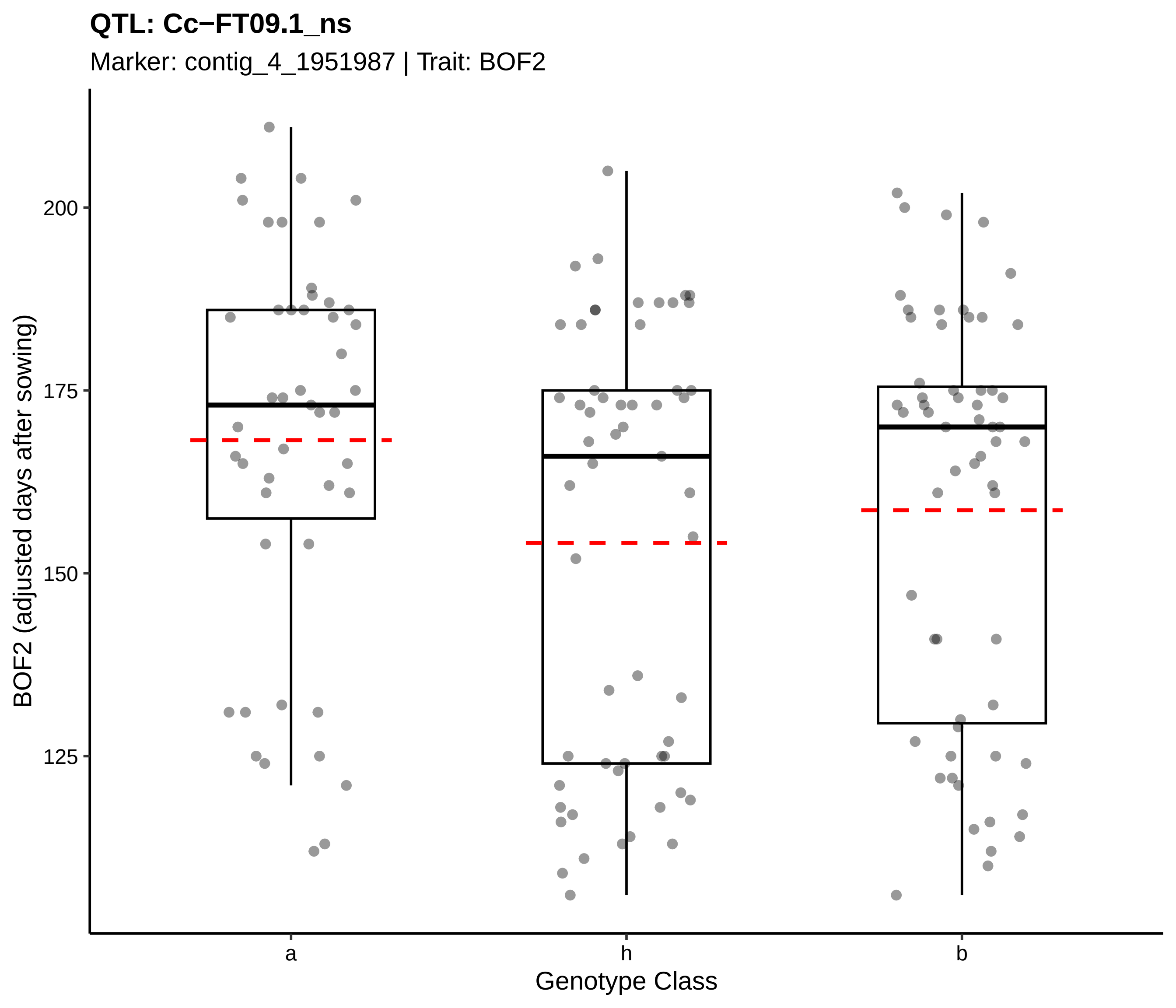
QTL: Cc−FT09.1_ns
Marker: contig_4_1951987 | Trait: BOF2
BOF2 (adjusted days after sowing)
200
175
150
125
a
h
b
Genotype Class

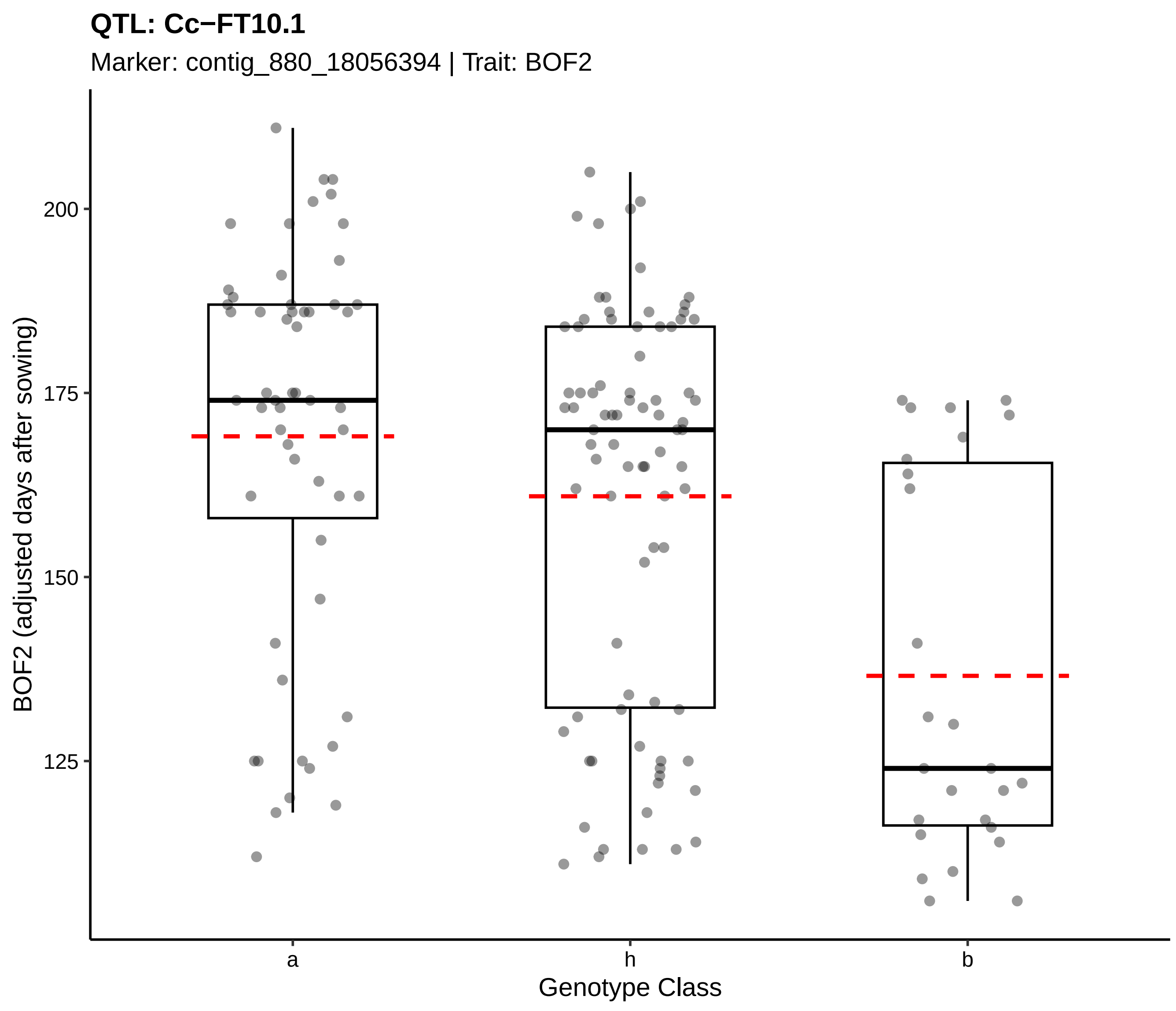

QTL: Cc−FT10.1
Marker: contig_880_18056394 | Trait: BOF2
BOF2 (adjusted days after sowing)
200
175
150
125
a
h
b
Genotype Class

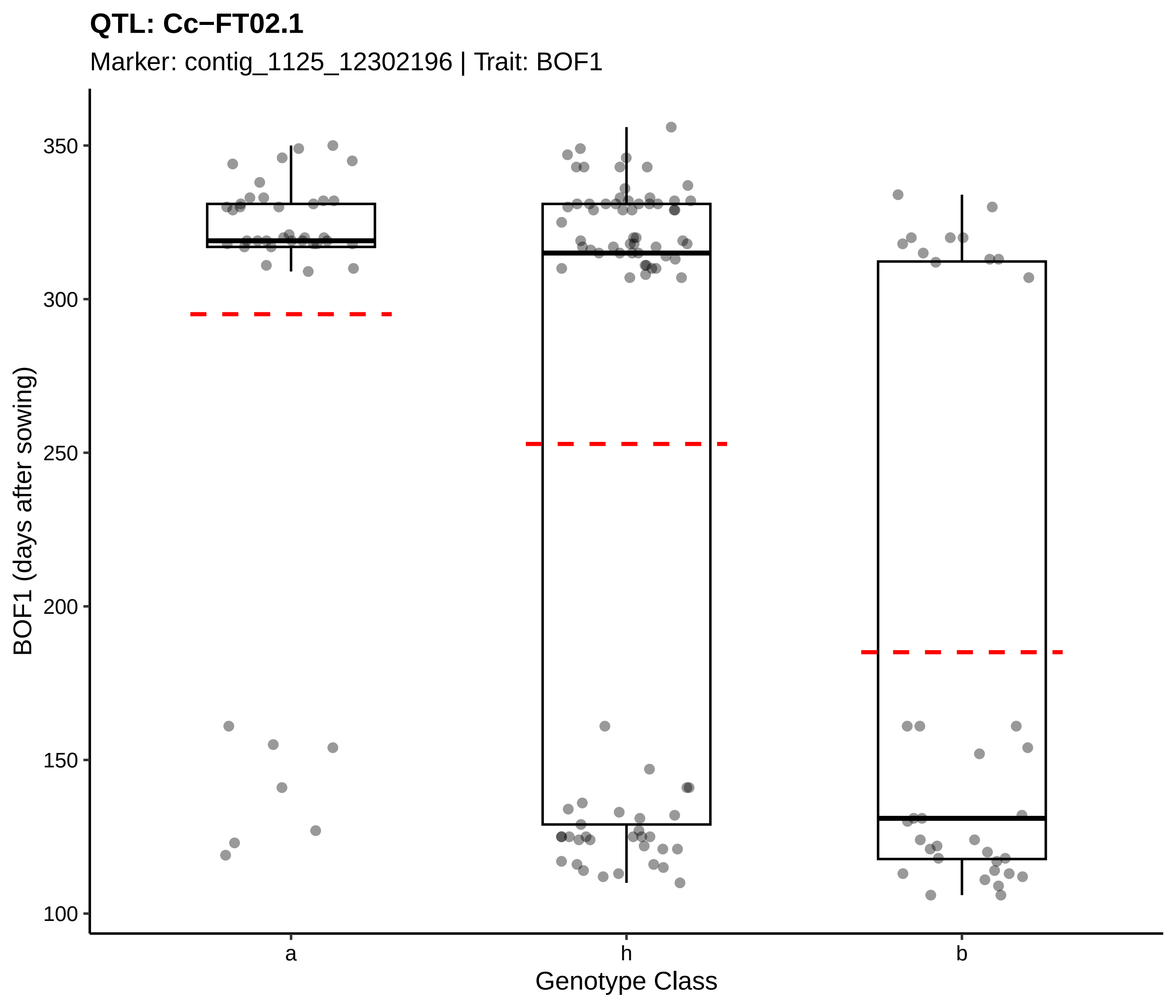
QTL: Cc−FT02.1
Marker: contig_1125_12302196 | Trait: BOF1
BOF1 (days after sowing)
350
300
250
200
150
100
a
h
b
Genotype Class

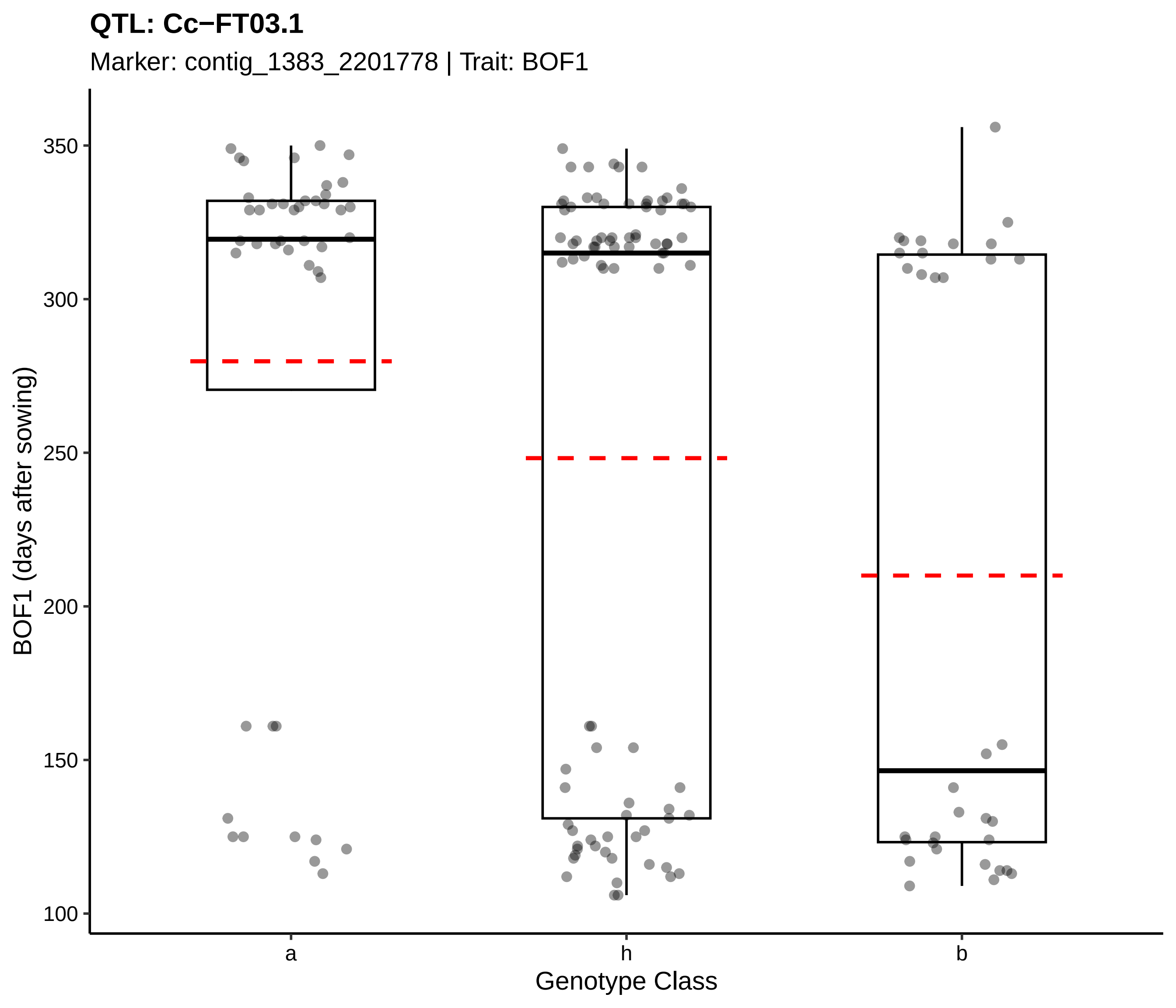
QTL: Cc−FT03.1
Marker: contig_1383_2201778 | Trait: BOF1
BOF1 (days after sowing)
350
300
250
200
150
100
a
h
b
Genotype Class

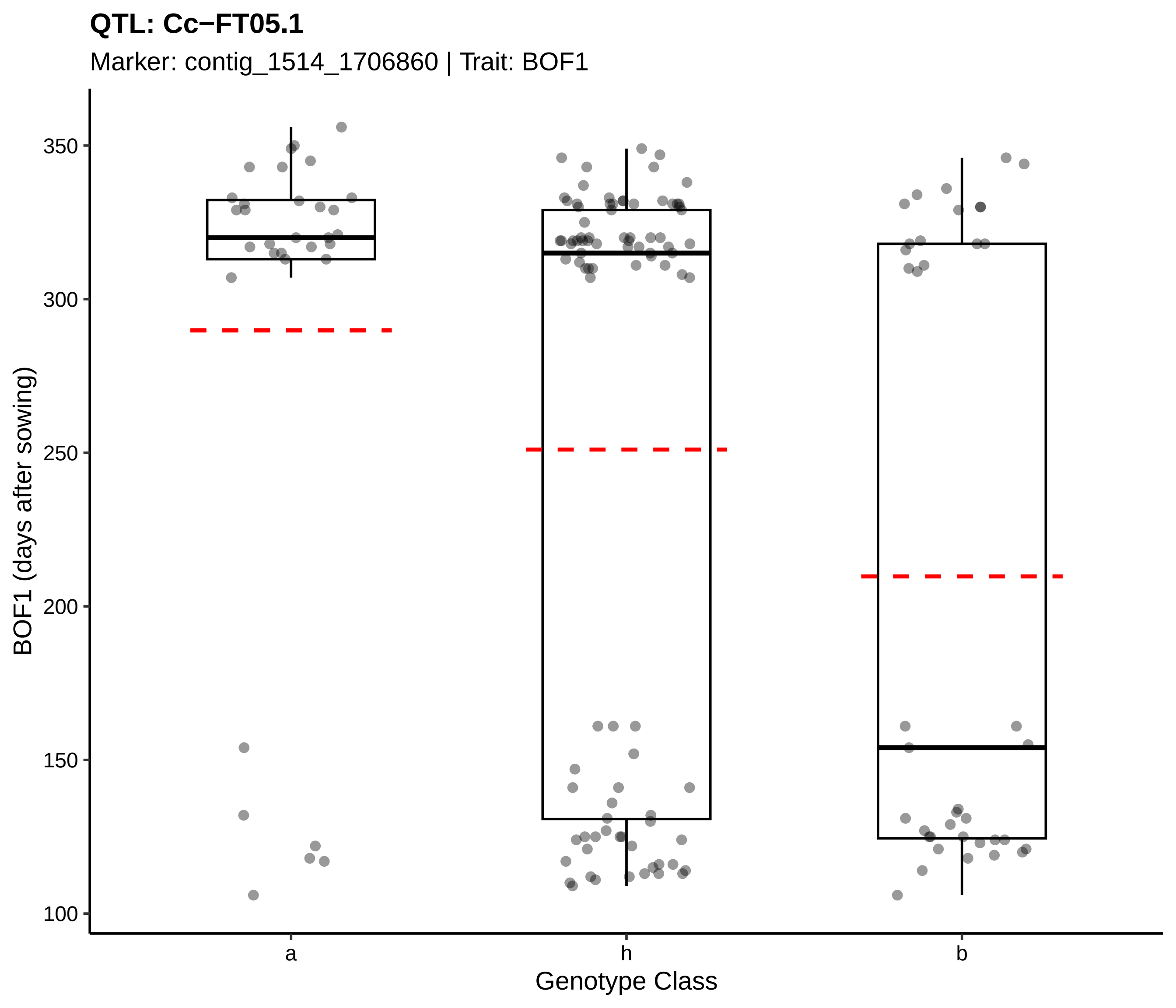
QTL: Cc−FT05.1
Marker: contig_1514_1706860 | Trait: BOF1
BOF1 (days after sowing)
350
300
250
200
150
100
a
h
b
Genotype Class

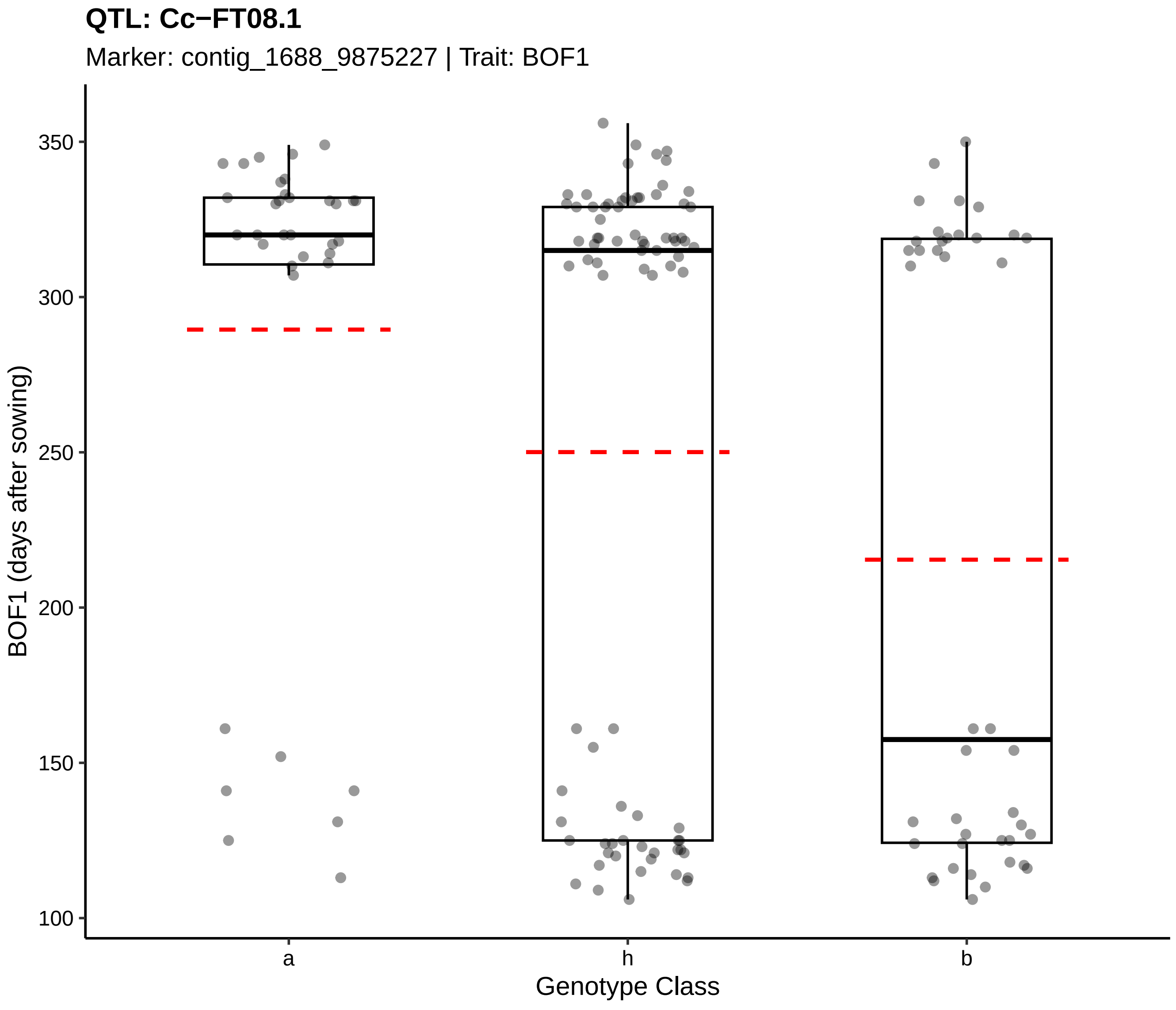
QTL: Cc−FT08.1
Marker: contig_1688_9875227 | Trait: BOF1
BOF1 (days after sowing)
350
300
250
200
150
100
a
h
b
Genotype Class

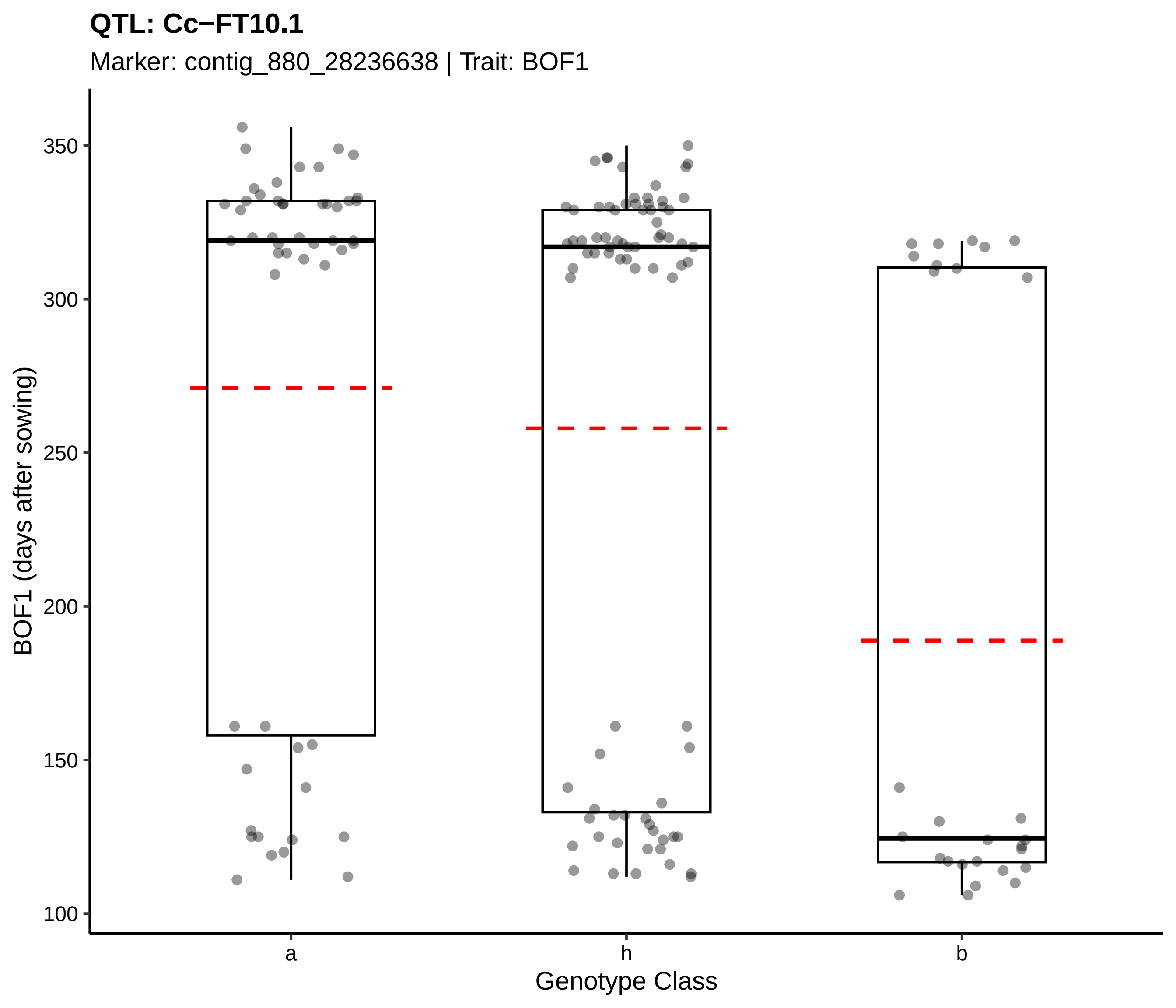
QTL: Cc−FT10.1
Marker: contig_880_28236638 | Trait: BOF1
BOF1 (days after sowing)
350
300
250
200
150
100
a
h
b
Genotype Class

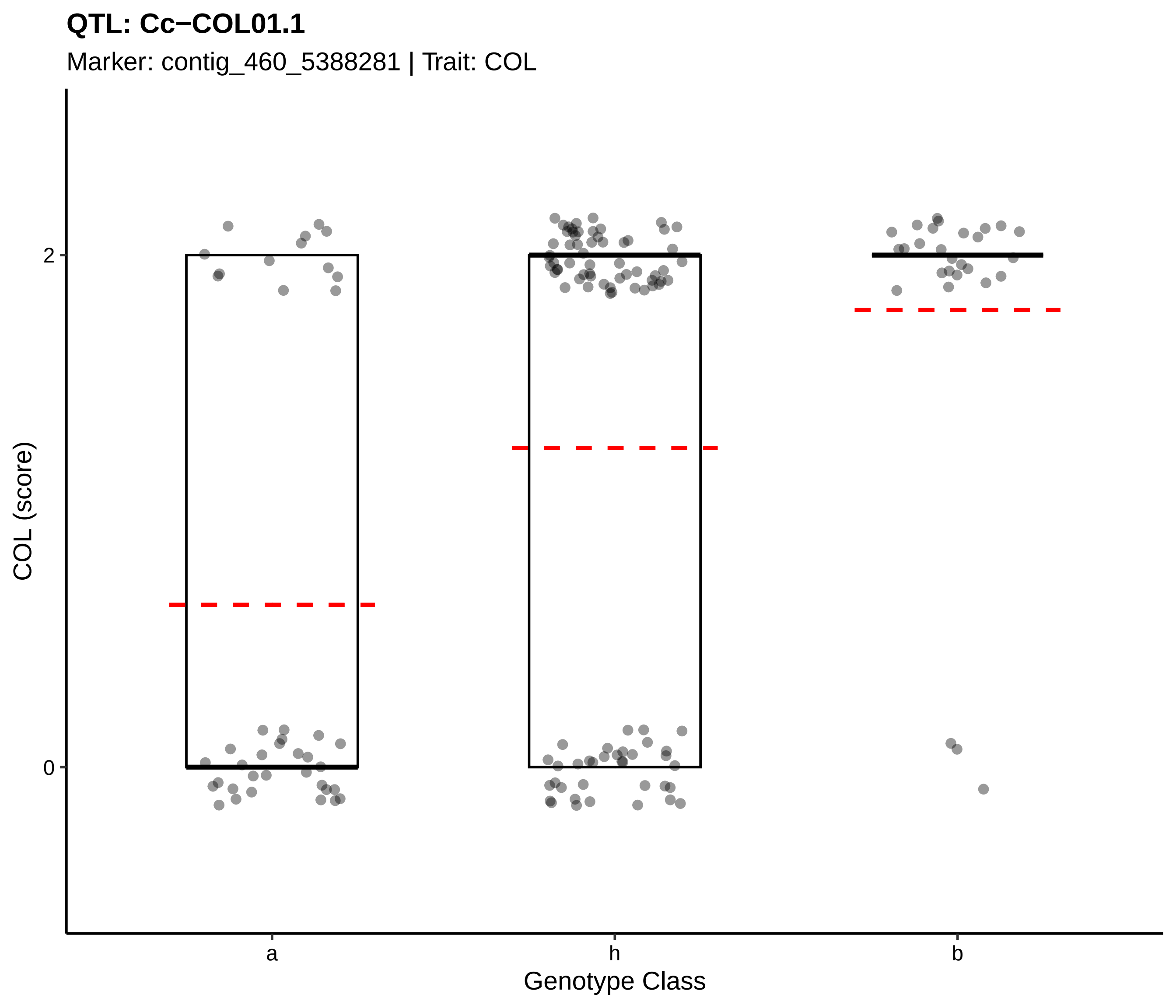
QTL: Cc−COL01.1
Marker: contig_460_5388281 | Trait: COL
COL (score)
2
0
a
h
b
Genotype Class

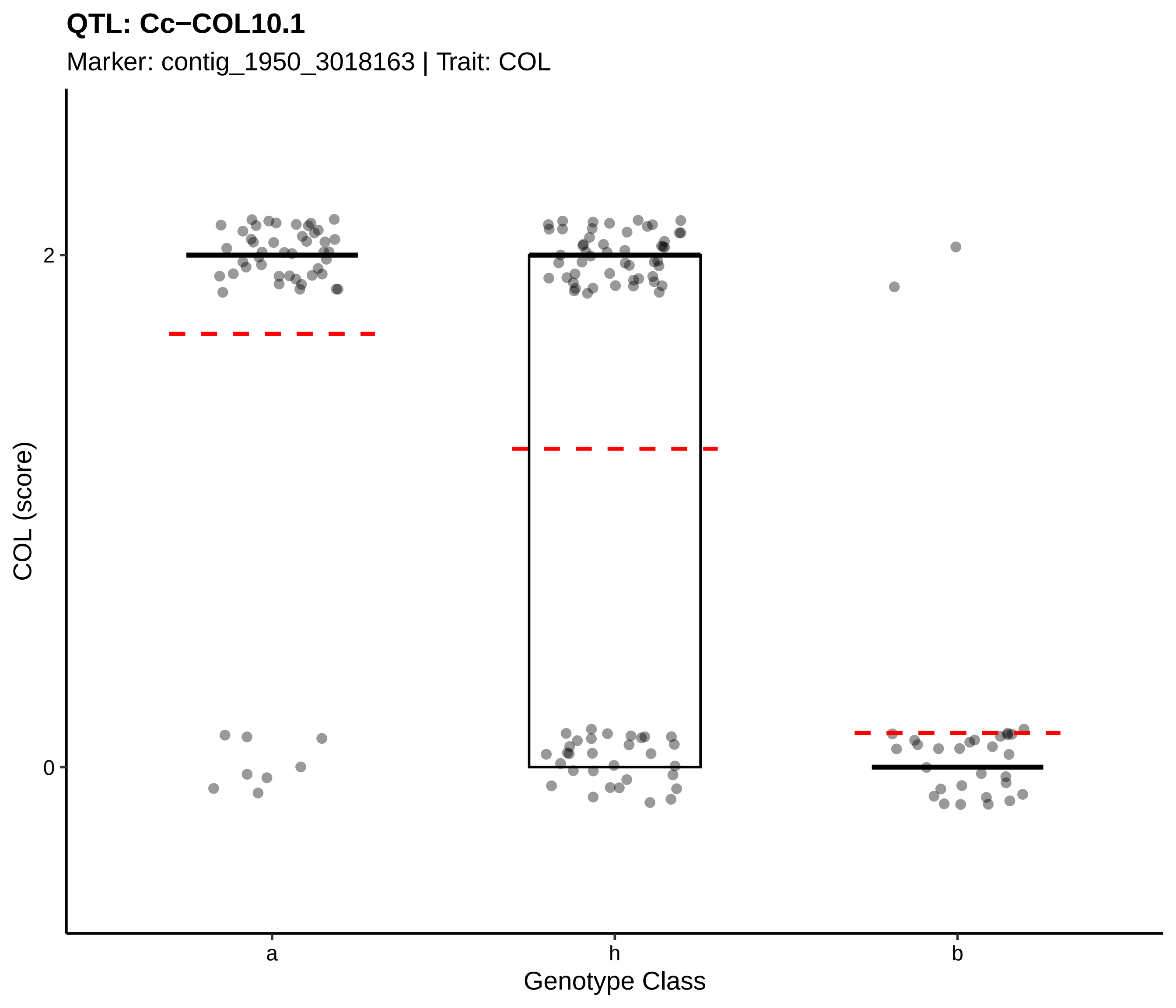
QTL: Cc−COL10.1
Marker: contig_1950_3018163 | Trait: COL
COL (score)
2
0
a
h
b
Genotype Class

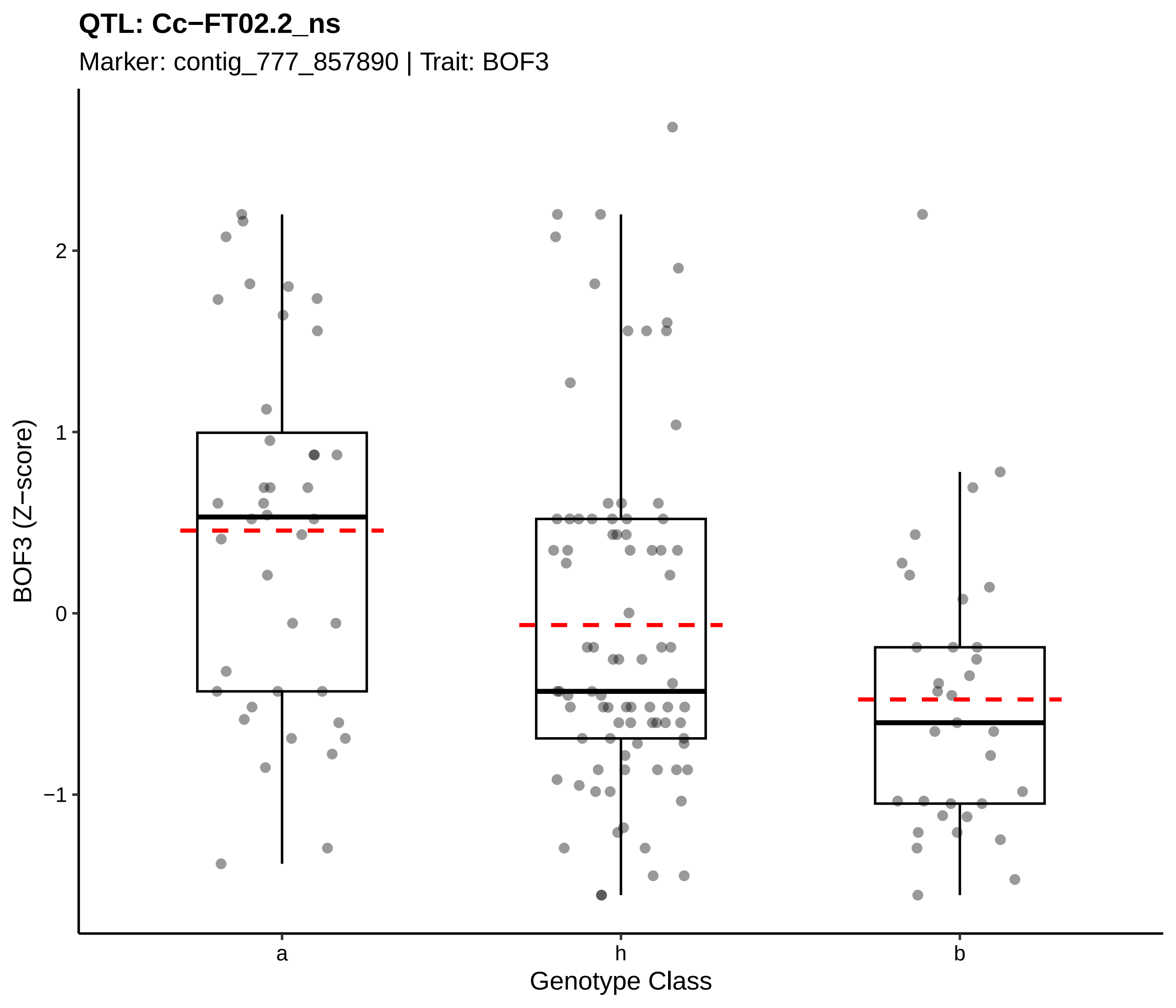

QTL: Cc−FT02.2_ns
Marker: contig_777_857890 | Trait: BOF3
BOF3 (Z−score)
2
1
0
−1
a
h
b
Genotype Class

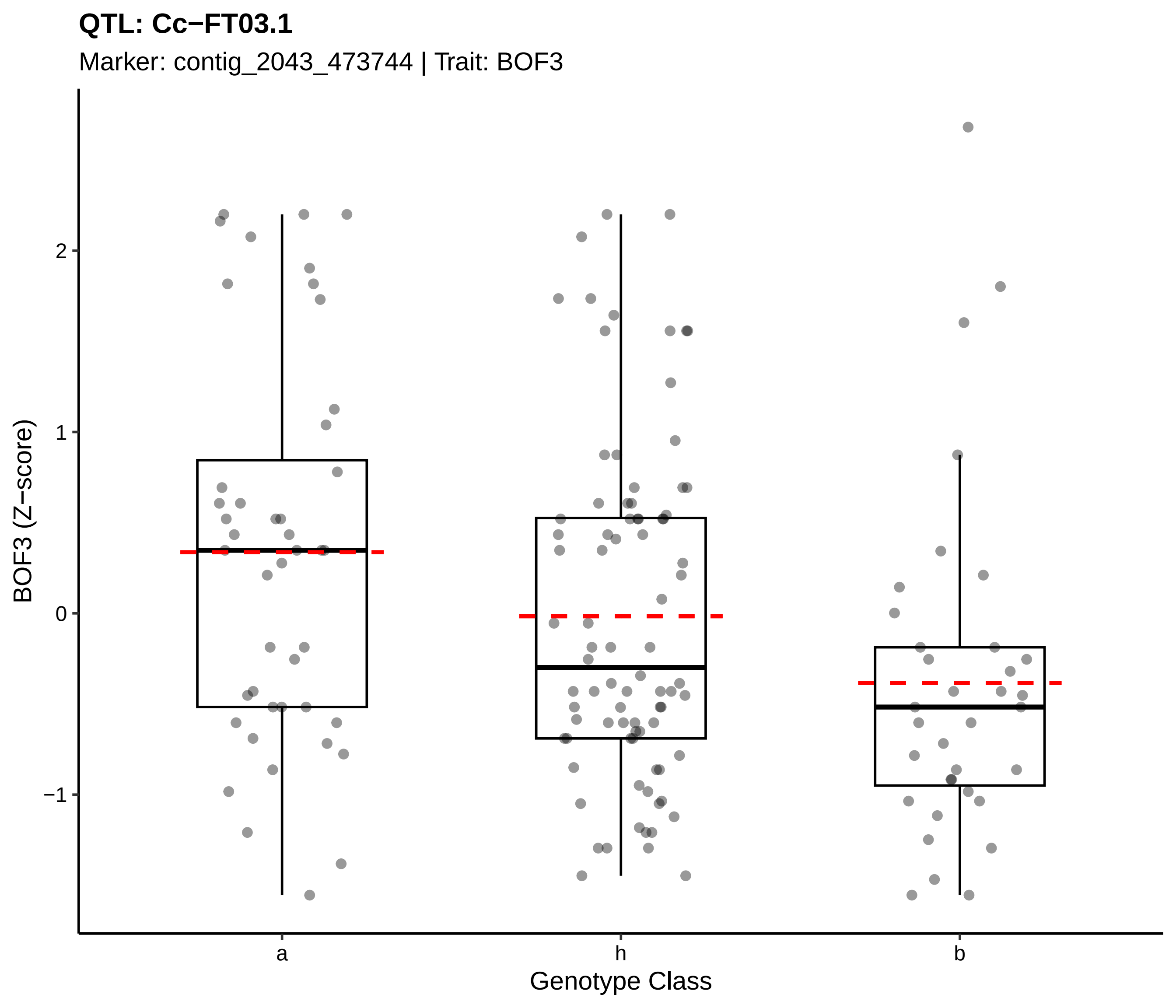
QTL: Cc−FT03.1
Marker: contig_2043_473744 | Trait: BOF3
BOF3 (Z−score)
2
1
0
−1
a
h
b
Genotype Class

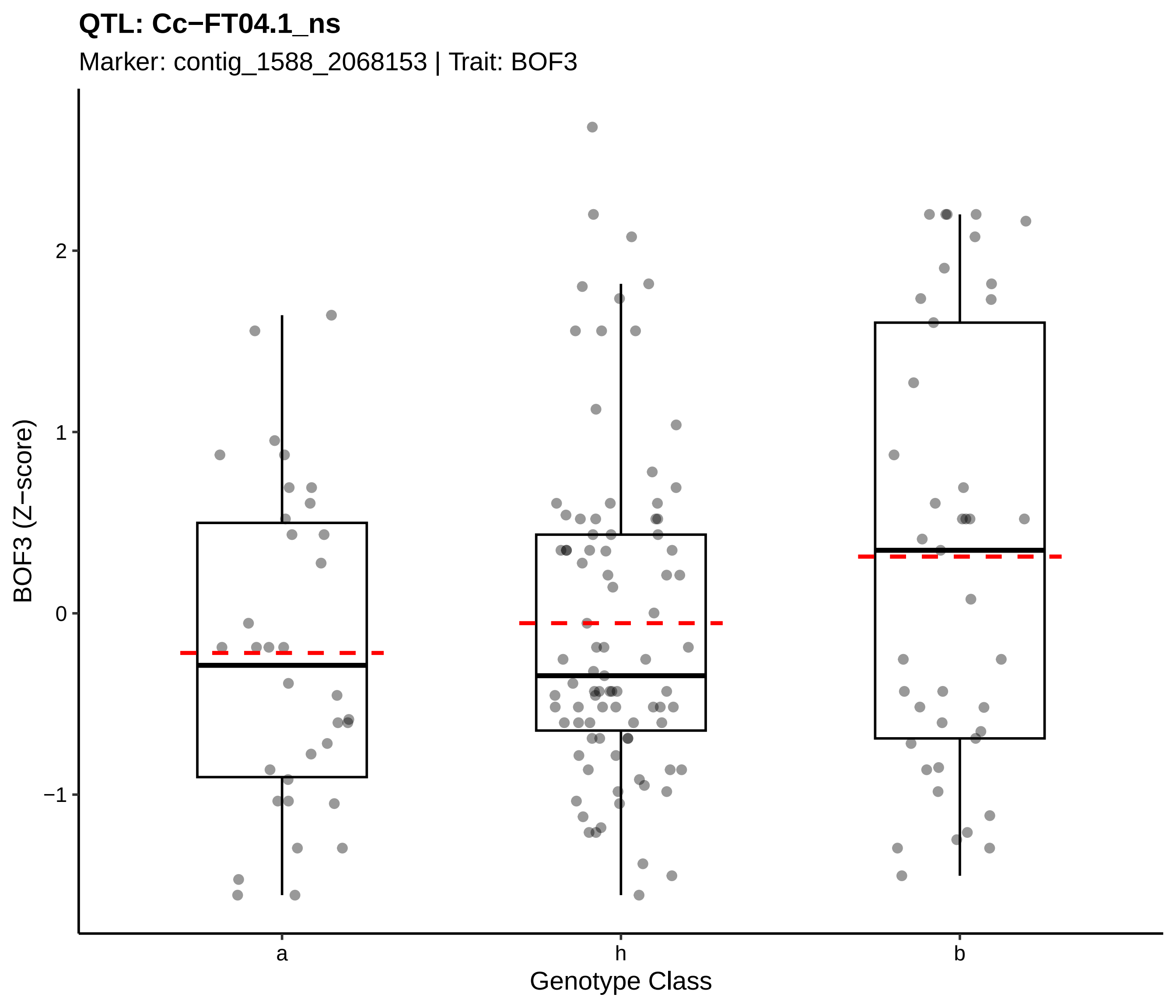
QTL: Cc−FT04.1_ns
Marker: contig_1588_2068153 | Trait: BOF3
BOF3 (Z−score)
2
1
0
−1
a
h
b
Genotype Class

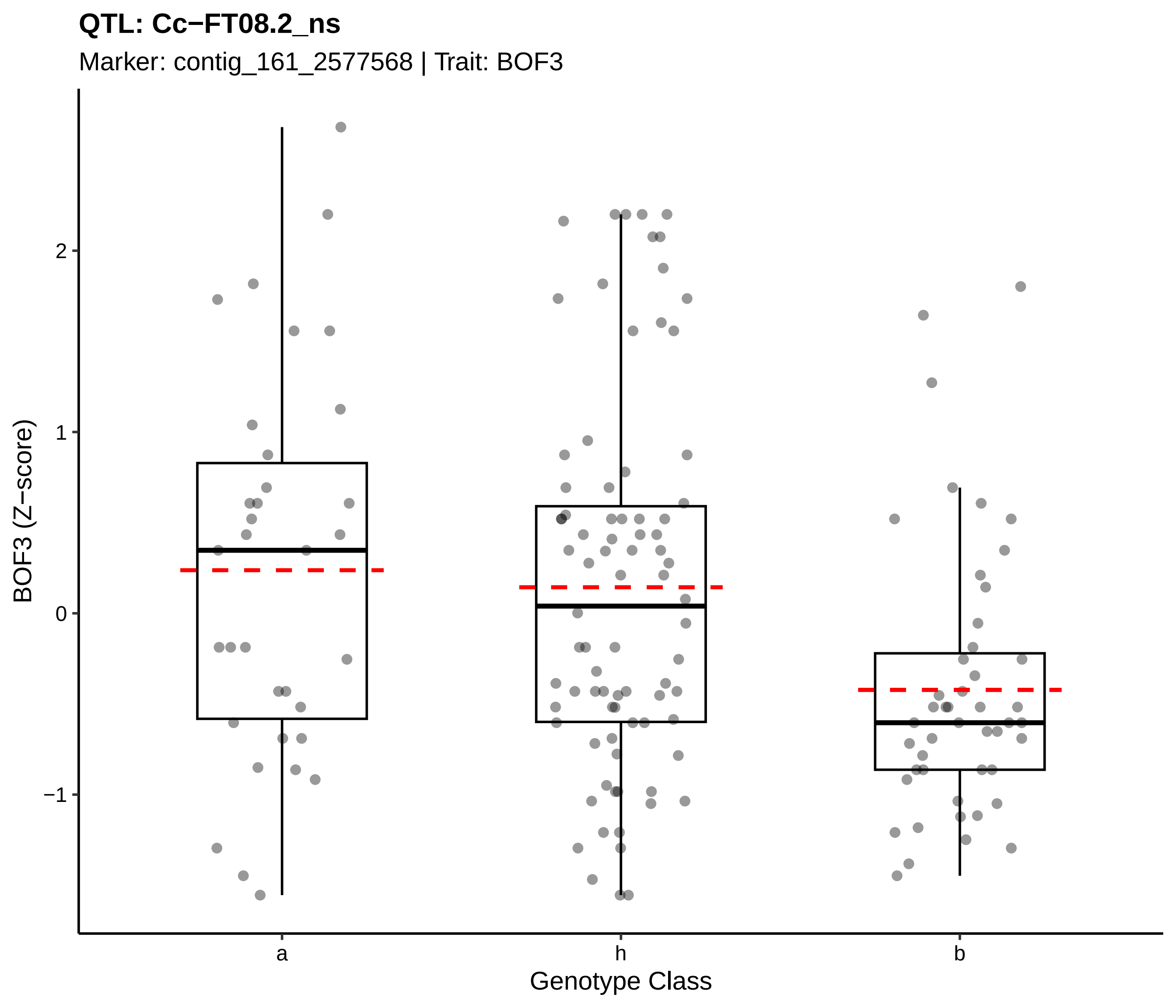

QTL: Cc−FT08.2_ns
Marker: contig_161_2577568 | Trait: BOF3
BOF3 (Z−score)
2
1
0
−1
a
h
b
Genotype Class

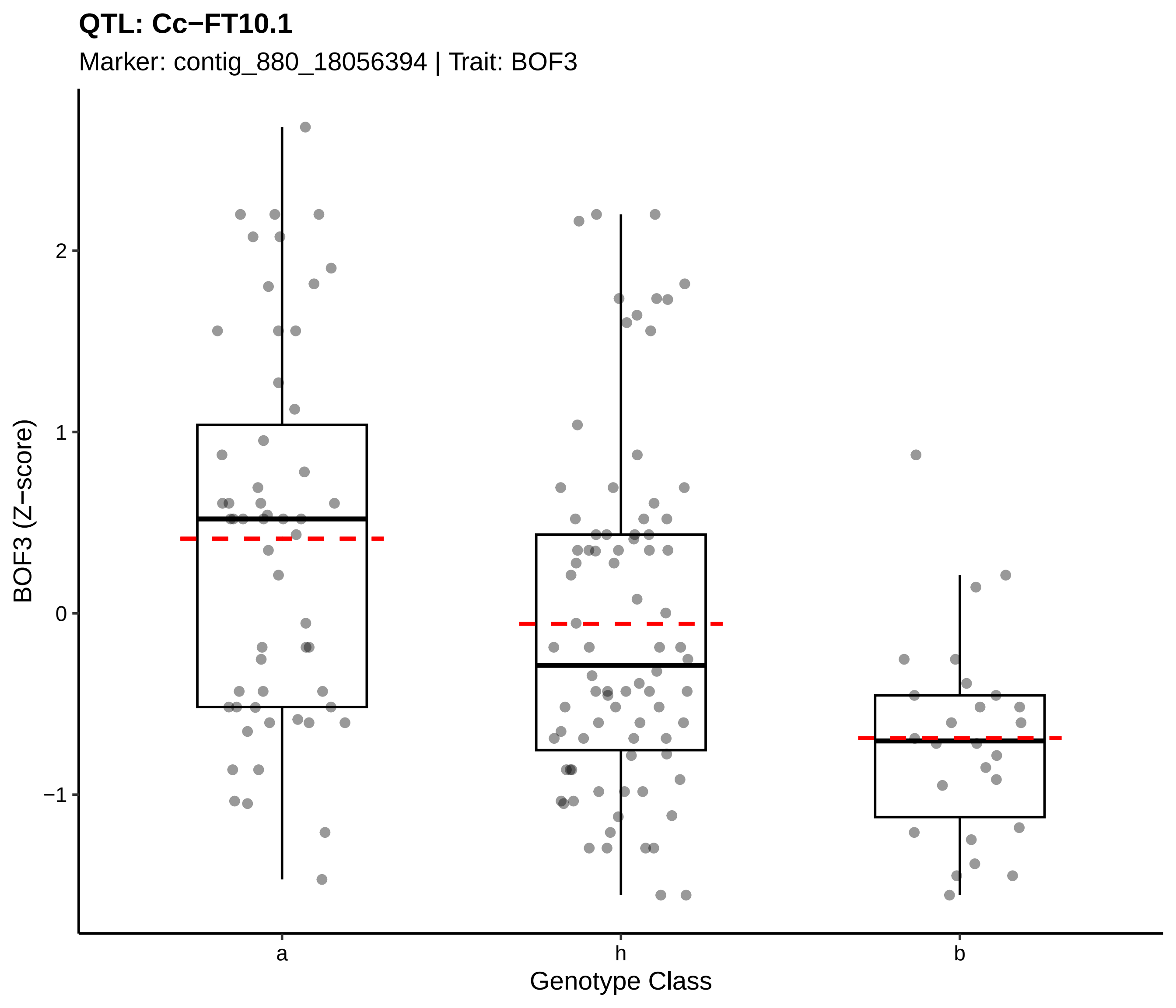

QTL: Cc−FT10.1
Marker: contig_880_18056394 | Trait: BOF3
BOF3 (Z−score)
2
1
0
−1
a
h
b
Genotype Class
